# Systemically delivered mRNA-LNPs transfect primary and secondary liver tumors

**DOI:** 10.1016/j.omtn.2026.102989

**Published:** 2026-06-17

**Authors:** Laura J. Leighton, Yee Jing Gee, Sachithrani U. Madugalle, Maria Victorova, Nissa L. Carrodus, Kim R. Bridle, Sidney A. Howell, Xiaowen Liang, Gregory C. Miller, Chris L.D. McMillan, Danushka K. Wijesundara, David A. Muller, Darrell H.G. Crawford, Timothy R. Mercer, Seth W. Cheetham

**Affiliations:** 1 Australian Institute for Bioengineering and Nanotechnology, The University of Queensland, Brisbane, QLD, Australia; 2 National Biologics Facility, The University of Queensland, Brisbane, QLD, Australia; 3 BASE mRNA Facility, The University of Queensland, Brisbane, QLD, Australia; 4 Faculty of Health, Medicine and Behavioural Sciences, The University of Queensland, Brisbane, QLD, Australia; 5 Gallipoli Medical Research, Brisbane, QLD, Australia; 6 The University of Queensland, Brisbane, QLD, Australia; 7 The University of Queensland Frazer Institute, Brisbane, QLD, Australia; 8 Envoi Pathology, Brisbane, QLD, Australia; 9 School of Chemistry and Molecular Biosciences, The University of Queensland, Brisbane, QLD, Australia

**Keywords:** MT: Delivery Strategies, mRNA therapeutics, liver cancer, RNA delivery, mRNA-LNPs, lipid nanoparticles, SM-102, hepatocellular carcinoma, liver fibrosis, liver metastasis

## Abstract

Primary liver cancer is the sixth most prevalent cancer globally and is often diagnosed late, when treatment options are limited. Secondary liver cancer, arising from metastasis of other cancers to the liver, is a common complication of advanced solid cancers and a significant cause of cancer-related morbidity and mortality. Existing treatment options for advanced primary and secondary liver tumors have limited efficacy, and new treatment modalities have the potential to improve patient outcomes. Messenger RNA (mRNA) therapeutics are readily delivered to the healthy liver after systemic administration, but their uptake and expression within liver tumors are unclear. Here, we show that intravenous delivery of mRNA lipid nanoparticles (LNPs) efficiently transfects virtually all hepatocytes in healthy, fibrotic, and cirrhotic liver and also many cells of spontaneous hepatocellular carcinomas *in situ*. Delivery of mRNA is also possible in xenograft models of both primary and secondary liver cancer, albeit with attenuated protein expression relative to the normal liver. These findings demonstrate the potential for systemically delivered mRNA-LNP therapies for liver disease and cancer.

## Graphical abstract

**Figure:**
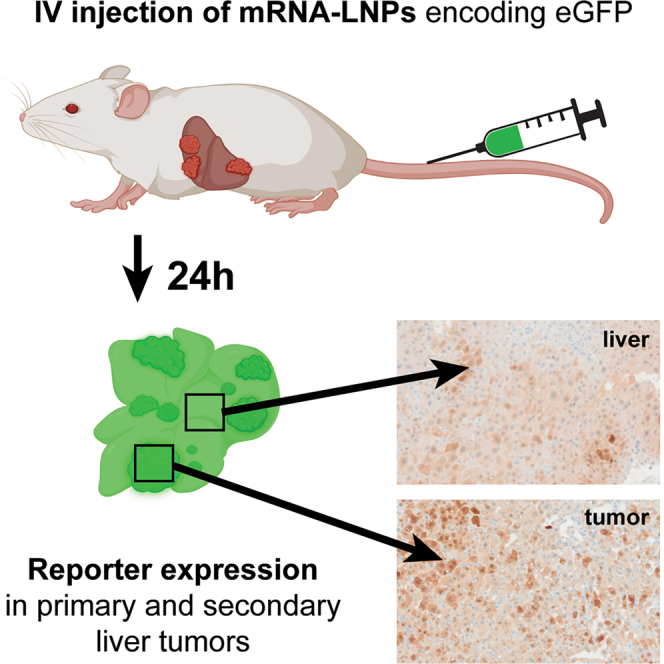

## Teaser

Messenger RNA formulated in LNPs effectively transfects primary and secondary liver cancers after intravenous injection. Strong delivery to spontaneous hepatocellular carcinomas is observed in the Mdr2 knockout mouse model and moderate delivery to tumors derived from xenografted human cancer cells.

## Introduction

Primary liver cancer is the sixth most prevalent cancer worldwide and the third-leading cause of cancer mortality.^1^ Around 75% of primary liver cancers are hepatocellular carcinomas (HCCs), which arise from malignant transformation of hepatocytes, the most common cell type in the liver. Primary liver cancer arises predominantly from chronic liver disease. Liver injury arising from chronic viral hepatitis, excessive alcohol use, and metabolic dysfunction-associated liver disease (MASLD) results in progressive fibrosis and ultimately cirrhosis, which predisposes individuals to HCC.^2^^,^^3^ The increasing prevalence of MASLD in developed countries is driving increased incidence of primary liver cancer.^3^

When detected early, primary liver cancer can be cured by surgical resection or liver transplantation, but for patients with advanced disease, treatment options are limited and often burdensome (reviewed by Liu et al.^4^). Transarterial chemoembolization (TACE) and selective internal radiation therapy (SIRT) deliver therapeutics directly to the tumor while also interrupting its blood supply.^5^^,^^6^^,^^7^^,^^8^^,^^9^ Systemic drug therapies are typically used in advanced HCC, in particular sorafenib and lenvatinib (inhibitors of protein kinases including VEGFR), and the combination of atezolizumab (a PD-L1 inhibitor) and bevacizumab (which targets VEGF-A).^9^^,^^10^^,^^11^^,^^12^ These systemic treatments can extend survival for several months on average but are rarely curative and associated with burdensome side effects including cutaneous reactions, stomatitis, peripheral neuropathy, gastrointestinal symptoms, and fatigue. The prognosis for primary liver cancer remains poor, with 5-year survival of approximately 22%.^13^^,^^14^

Secondary liver cancer, caused by metastasis of other cancers to the liver, is a substantial contributor to cancer morbidity and mortality. Around 50% of patients with metastatic colorectal, breast, and pancreatic cancer, and around 35% of patients with metastatic lung cancer, develop liver metastases,^15^^,^^16^^,^^17^^,^^18^ and these liver tumors are a frequent cause of cancer death.^15^^,^^17^^,^^18^^,^^19^^,^^20^ For secondary liver cancer patients also, treatment options are limited and include liver resection, ablative techniques, embolization therapies, and systemic chemotherapy or immunotherapy.^21^^,^^22^^,^^23^ However, secondary liver cancer generally carries a poor prognosis.^20^^,^^23^ Alternative treatment modalities are urgently needed for primary and secondary liver cancers.

The rapid development of SARS-CoV-2 vaccines powerfully demonstrated the safety and efficacy of synthetic messenger RNA (mRNA) medicines. Synthetic mRNAs comprise a coding sequence that can encode any therapeutic protein, flanked by 5′ and 3′ untranslated regions from highly expressed and stable mammalian mRNAs, and a poly(A) tail. To attenuate the innate response to foreign RNA,^24^ mRNA drugs are depleted of secondary structures and uridine residues, and the remaining uridines are substituted with the uridine analog N1-methylpseudouridine. Formulation of mRNA into lipid nanoparticles (LNPs) protects the mRNA and enables delivery to cells via endocytosis. A small percentage of the delivered mRNA escapes the endosome into the cytoplasm, where it is then translated by cytoplasmic ribosomes to produce the target protein. As of 2023, over 13 billion doses of mRNA vaccines had been administered globally with an adverse-event rate comparable to other vaccines,^25^ demonstrating the fundamental effectiveness and safety of this drug modality.

Like most nanoparticles in the 50–250 nm size range,^26^ intravenously injected mRNA-LNPs accumulate predominantly in the liver following intravenous injection, and the mRNA-encoded protein is expressed by hepatocytes for several days^27^; some LNP formulations show preferential expression in other organs.^28^ Hepatic mRNA uptake encompasses virtually all hepatocytes and subpopulations of endothelial and Kupffer cells.^29^ The natural tissue tropism of LNPs for hepatocytes has enabled experimental mRNA-based treatments of genetic metabolic disorders including methylmalonic acidemia,^30^ acute intermittent porphyria,^31^ and Fabry disease^32^ in animal models, and a systemically administered mRNA-LNP treatment for propionic acidemia has shown promise in early human trials.^33^ Importantly, these studies have demonstrated the safety and efficacy of repeated mRNA-LNP administration, which is critical if mRNA drugs are to be used for ongoing treatment of liver disease.

The efficient delivery of mRNA-LNPs to healthy livers raises the possibility of using mRNA therapeutics for the treatment of liver disease and cancer. Primary liver cancer typically arises on a background of chronic liver disease and hepatic fibrogenesis. One study reported good expression of mRNA-LNPs (formulated using an ionizable lipid proprietary to Acuitas) in hepatocytes in several mouse models of fibrosis and cirrhosis and a therapeutic effect of *Hnf4a* mRNA against fibrosis.^34^ However, the extent of transgene expression in liver tumors after systemic mRNA-LNP administration has never been rigorously assessed.

In this study, we investigated the distribution and intensity of reporter gene expression in multiple mouse models of liver disease and cancer following intravenous administration of mRNA-LNPs based on the ionizable lipid SM-102 (molar ratios: 50 SM-102/10 DSPC/38.5 cholesterol/1.5 DMG-PEG2000; see materials and methods). SM-102 is among the most extensively characterized ionizable lipids in the preclinical mRNA literature,^35^ facilitating direct comparison with existing and future studies, and also has FDA approval for clinical use. SM-86, a close structural analog of SM-102, has been used in human clinical trials, targeting the liver by intravenous infusion.^33^

Here, we show that following intravenous administration, mRNA-LNPs enable efficient protein expression not only in healthy liver but also in fibrotic and cirrhotic liver, spontaneous HCCs *in situ*, and xenograft models of primary and secondary liver cancer in mice. These findings suggest that intravenous injection of mRNA-LNPs without a specific targeting moiety can achieve widespread transgene expression, supporting the further development of mRNA-LNP therapeutics for liver disease and cancer.

## Results

### Efficient delivery of mRNA-LNPs to the healthy mouse liver

**Figure 1:**
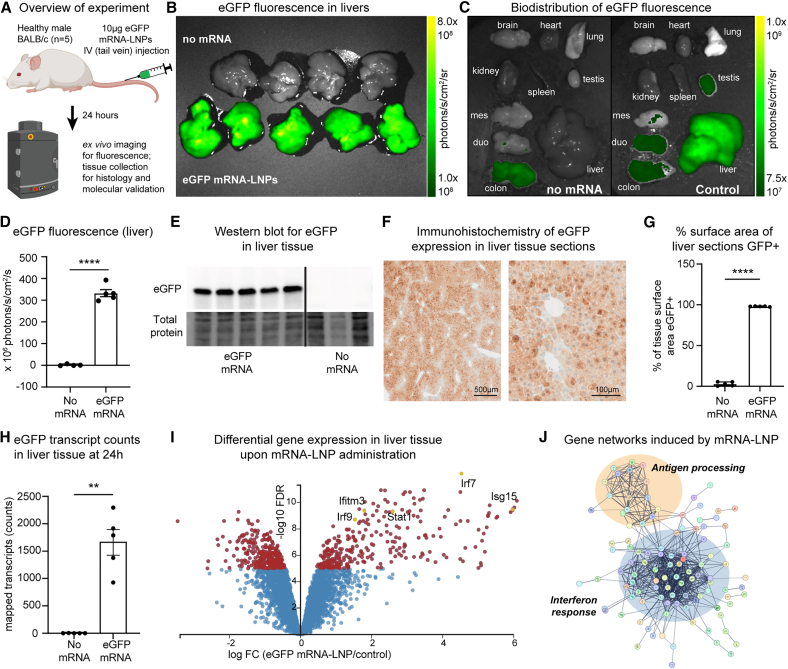
Efficient delivery of mRNA-LNPs to the healthy mouse liver (A) We injected 5 healthy mice with 10 μg eGFP mRNA-LNPs via the tail vein and investigated reporter expression after 24 h. (B) Strong eGFP fluorescence was visualized in all injected livers (bottom row). (C) *Ex vivo* fluorescence imaging of organs from injected and uninjected mice shows specificity of eGFP expression in the liver. Fluorescence scales were set to eliminate autofluorescent signal in the uninjected liver; autofluorescent signal is visualized in the colon and duodenum of both the injected and uninjected mouse. (D) Quantitation of background-subtracted eGFP fluorescence confirms the strong and specific detection of eGFP in the livers of eGFP-injected mice (Welch’s *t* test, *n* = 4–5, t = 20.25(4.117), *p* < 0.0001). Error bars represent mean ± SEM. (E) Western blotting detects a strong band corresponding to eGFP in the livers of mice injected with eGFP mRNA-LNPs; no band is detected in liver tissue of uninjected mice. (F) Immunohistochemistry for eGFP in liver tissue sections from injected mice shows strong eGFP staining in virtually all hepatocytes. There is moderate variation in staining intensity between hepatocytes. (G) Quantification of GFP-positive surface area for IHC sections in GFP-injected mice shows extremely high coverage of liver tissue, with very low background in uninjected mice (Welch’s *t* test, *n* = 5, t = 84.81(4.204), *p* < 0.0001). Error bars represent mean ± SEM. (H) eGFP transcripts were readily detected in mouse liver tissue 24 h after injection, with mapped transcript counts between approximately 1,000 and 2,300; this was significantly different from control with an average of one count (Mann-Whitney test, *n* = 5, U = 0, *p* = 0.0079). Error bars represent mean ± SEM. (I) 624 genes were differentially expressed in the liver between mRNA-LNP-injected and saline-injected mice, with the significance threshold of 5 ∗ −log10 FDR. These included interferon response factors (*Irf7*, *Irf9*, *Ifitm3*, and *Isg15*) and the cytokine-responsive transcription factor *Stat1*. (J) Gene networks induced in the liver by mRNA-LNP injection; upregulated genes cluster into antigen-processing and interferon-response networks. Figure partially created in BioRender. Leighton, L. (2026) https://BioRender.com/y17kh8g.

We initially set out to establish a baseline for mRNA-LNP delivery to and expression in the mouse liver. We selected a formulation for mRNA-LNPs based on the ionizable lipid SM-102, which is approved for clinical use. To assess the delivery of mRNA to the liver, we intravenously injected adult male mice (*n* = 5) with mRNA-LNPs encoding enhanced green fluorescent protein (eGFP) (Figure 1A). Strong and specific eGFP fluorescence was observed in the liver after 24 h via *ex vivo* fluorescence imaging, with measured fluorescence values around 10-fold higher than background (tissue autofluorescence) (Figures 1B and 1C). Background-subtracted fluorescence (radiant efficiency) of whole livers was significantly different between mRNA-injected and uninjected mice (Student’s *t* test, *n* = 4–5, *p* < 0.0001) (Figure 1D). Western blotting detected eGFP protein strongly from the liver tissue of these mice (Figure 1E), and in one healthy mouse from which additional organs were blotted, eGFP was also detected in the spleen (Figure S1). Notably, green fluorescent signal in the digestive tract likely arises from gut contents^36^ and does not indicate the presence of eGFP (Figure S1). eGFP was not detected in serum from any of 6 injected mice tested (Figure S2), confirming that eGFP is not secreted into the bloodstream and that no spurious signal is expected to arise from blood.

In the livers of injected mice, virtually all hepatocytes expressed eGFP mRNA as evident from immunohistochemistry (IHC) (Figures 1F and S3), with variable staining intensity between cells and no obvious bias in spatial distribution. This demonstrated the efficient uptake and expression of mRNA by the healthy liver and provided a basis for comparison of the results in diseased liver.

To quantify the distribution of eGFP expression in tissue sections, we trained a machine learning algorithm, *StainDetectAI*, to distinguish stained from unstained liver tissue in IHC microscopy images (Figures S4 and S5). We chose to use machine learning rather than conventional rule-based image analysis software because it enabled us to perform rapid, unbiased, and automated analysis using the full dataset rather than a subsample. The model was trained using a supervised machine learning approach, using a training dataset consisting of manually annotated IHC images of GFP-positive and GFP-negative liver tissue selected from the IHC images generated during this study. The model has a specificity of 98% and sensitivity of 92%, with an overall prediction accuracy of 95%. Using *StainDetectAI*, we showed that eGFP mRNA-LNP-injected animals had around 97% of liver section surface area stained for eGFP (Figure 1G), while on average less than 3% of surface area was falsely detected as stained in liver sections from uninjected mice. The surface area detected as stained between injected and uninjected mice was significantly different (Welch’s *t* test, *n* = 5, *p* < 0.0001).

An important consideration for the safety and efficacy of mRNA-LNP therapeutics delivered via the intravenous route is the induction of an immune response, and potentially other off-target gene expression, in the liver. To determine whether the mRNA-LNP platform impacts on liver gene expression, we sequenced RNA from liver tissue of healthy mice injected with eGFP mRNA-LNPs (*n* = 5) and saline-injected controls (*n* = 5) 24 h after injection. We detected abundant eGFP transcripts (averaging 1,661 counts) in the livers of mRNA-injected mice, with negligible background (average 1 count in uninjected mice), demonstrating that mRNA can persist in liver tissue for at least a day after injection (Figure 1H); the difference was significant (Mann-Whitney test, *n* = 5, *p* = 0.0079). Consistent with the known immunogenicity of mRNA-LNPs, particularly those formulated with SM-102,^37^ intense upregulation of genes involved in innate immunity was observed in the liver tissue of mRNA-injected mice relative to uninjected controls, including *Irf7/Irf9*, *Stat1*, and *IfitmFITM3*. In total, 295 genes were significantly upregulated and 323 genes significantly downregulated (Figure 1I; Table S1; Figure S6). Notably, PD-L1 (*Cd274*) was significantly upregulated in liver cells following mRNA-LNP treatment (Welch’s *t* test, *n* = 5, *p* < 0.0001), substantiating recent reports that mRNA-LNP administration can increase checkpoint ligand expression. The widespread immune response is consistent with previous reports describing inflammatory responses to mRNA-LNP delivery,^37^^,^^38^ underscoring the importance of carefully controlling for platform effects when evaluating candidate therapeutics.

### Delivery of mRNA-LNPs in liver fibrosis and cirrhosis

**Figure 2:**
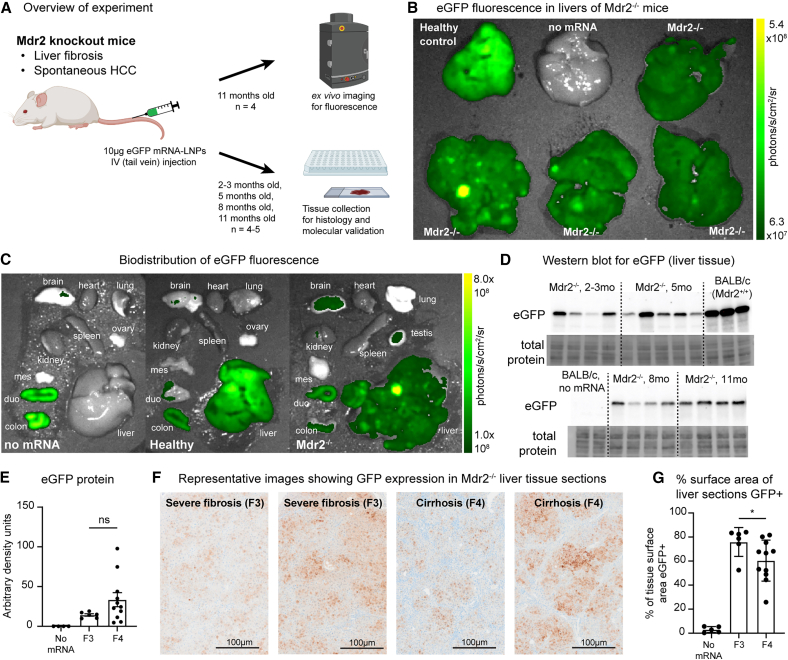
Delivery of mRNA-LNPs in liver fibrosis and cirrhosis (A) We injected *Mdr2*^−/−^ mice with 10 μg eGFP mRNA-LNPs via the tail vein and investigated reporter expression. (B) Moderately strong eGFP fluorescence was evident in all injected livers; the fluorescence intensity was reduced compared to a healthy control injected with the same mRNA-LNPs at the same time. (C) *Ex vivo* fluorescence imaging of organs from a GFP-injected *Mdr2*^−/−^ mouse shows that attenuation of mRNA-LNP delivery to the liver does not result in broadly altered biodistribution of eGFP protein expression. Autofluorescent signal was detected from the brain and gut tissues of all mice including healthy and uninjected controls. (D) Western blotting detects eGFP from the liver tissue of 17 *Mdr2*^−/−^ mice, with substantial variation between individuals in the quantity of eGFP protein and reduced protein abundance relative to healthy BALB/c mice. (E) Quantification of eGFP protein expression from western blotting showing greater variability in protein expression for *Mdr2*^−/−^ mice with a fibrosis score of F4 (cirrhosis) relative to F3 (severe fibrosis). The total protein abundance in F4 mice trended higher, but this difference was not statistically significant (Welch’s *t* test, *n* = 6–11, t = 2.162(10.58), *p* = 0.0545). Error bars represent mean ± SEM. (F) Representative immunohistochemistry results from *Mdr2*^−/−^ mice show the range of possible outcomes for eGFP expression. (G) Analysis of IHC images with *StainDetectAI* found that mice with severe fibrosis (F3) had detectable eGFP staining across a higher surface area compared to mice with cirrhosis (F4) (Mann-Whitney test, *n* = 6–11, U = 12, *p* = 0.0365). Error bars represent mean ± SEM. Figure partially created in BioRender. Leighton, L. (2026) https://BioRender.com/kbvlul3.

We next investigated delivery of mRNA-LNPs in a physiologically relevant mouse model of progressive liver injury. *Mdr2* knockout mice are unable to secrete phospholipids into the bile, resulting in the development of sclerosing cholangitis with severe liver fibrosis by early adulthood and spontaneous HCC by 1 year of age.^39^^,^^40^ We used the *Mdr2* knockout model to evaluate mRNA-LNP delivery to the liver in the context of liver fibrosis and cirrhosis (Figure 2A). We injected eGFP mRNA-LNPs intravenously into 4 male *Mdr2* knockout mice at 11 months of age, and 14 h post-injection, we observed fluorescent signal 3- to 4-fold above background in the liver tissue, albeit attenuated 2-fold relative to healthy controls (Figure 2B). Biodistribution of eGFP fluorescence in *Mdr2* knockout mice was comparable to that observed in healthy BALB/c mice (Figures 2C, S1, and S2), indicating that off-target expression of the mRNA in other organs and tissues is not increased in the presence of liver injury.

Separately, eGFP mRNA-LNPs were injected into male *Mdr2*^−/−^ mice aged 2–3 months (*n* = 4), 5 months (*n* = 4), 8 months (*n* = 5), and 11 months (*n* = 4). Using western blotting, we found that eGFP protein was evident in liver tissue from all mice after 24 h, with substantial variation between individuals; protein density ranged from 4.65 to 97.85 (arbitrary units, Figures 2D and 2E). Transfection of hepatocytes was widespread in most livers (Figures 2F and S7). However, the staining intensity and the proportion of transfected cells were both reduced relative to healthy livers (shown in Figure 1F), and staining was marginal to absent within fibrotic bands, demonstrating that extensive fibrosis reduces but does not ablate mRNA-LNP delivery efficiency. Using *StainDetectAI*, we found that the total surface area of liver tissue positive for GFP staining (quantified using *StainDetectAI*) ranged from 26% to 84% across all 17 mice.

Given the variability in disease severity between individual mice, we next evaluated eGFP mRNA expression relative to the severity of liver disease. We used the METAVIR scoring system to assess the extent of liver fibrosis based on H&E-stained liver tissue sections (Figure S7); out of 17 mice, 6 received a score of F3 (severe fibrosis) and 11 were scored F4 (cirrhosis). Importantly, we did not find a significant difference in eGFP protein expression between animals scoring F3 and F4 (Welch’s *t* test, *n* = 6–11, *p* = 0.0545) (Figure 2E). When comparing the result from *StainDetectAI* between F3 and F4 animals, we found that the stained surface area was significantly negatively associated with the degree of liver fibrosis (Figure 2G); animals with a fibrosis score of F3 averaged 76% surface area staining, while F4 animals averaged 60% (Mann-Whitney test, *n* = 6–11, *p* = 0.0365). One possible explanation for this result is that mice with more severe disease have more of the liver section surface area occupied by fibrotic bands, resulting in reduced hepatocyte surface area. We therefore quantified the stained surface area in 10 randomly selected liver tiles per animal in which any fibrotic areas were manually masked. This analysis found that in these hepatocyte-only samples, there remained a significant difference between animals with a fibrosis score of F3, which averaged 83% surface area staining, and animals scoring F4, which averaged 67% (Mann-Whitney test, *n* = 6–11, *p* = 0.0103) (Figure S8). This shows that the difference in distribution of mRNA expression between F3 and F4 animals is not merely a function of the increased surface area of fibrotic bands.

Several studies have shown that intravenously injected mRNA-LNPs based on clinically used ionizable lipids acquire a coating of apolipoprotein E (ApoE), which acts as a ligand for the low-density lipoprotein receptor (LDLR), and this is one pathway by which mRNA-LNPs are taken up by hepatocytes. To determine whether differential expression of LDLR could explain differential uptake of mRNA-LNPs between healthy and diseased liver tissue, we performed IHC for LDLR in healthy mice and *Mdr2*^−/−^ mice. Qualitatively, we observed a similar staining pattern and intensity between 3 healthy and 3 *Mdr2*^−/−^ livers, although notably, LDLR staining was absent from fibrotic bands (Figures S9A and S9B). To investigate the copy number of LDLR in healthy and diseased liver tissue, we drew on publicly available transcript abundance data from the GepLiver database^41^ (gepliver.org, accessed March 2026). In mice, *Ldlr* transcripts were significantly downregulated in fibrosis/cirrhosis compared to healthy liver (Kruskal-Wallis test with Dunn’s post hoc, *n* = 29–112, *p* = 0.0041.) However, in human data, *LDLR* copy number was increased in fibrotic liver (Kruskal-Wallis test with Dunn’s post hoc, *n* = 20–362, *p* < 0.0001) but unchanged in cirrhosis (Kruskal-Wallis test with Dunn’s post hoc, *n* = 73–262, *p* > 0.9999) (Figures S9C and S9D). These inconsistent findings suggest that any relationship between LDLR abundance and disease severity is likely to be species or context specific.

Overall, these results show that while fibrosis and cirrhosis reduce the efficiency of mRNA-LNP delivery to the liver, robust and widespread mRNA delivery is still evident, with the majority of hepatocytes transfected.

### Delivery of mRNA-LNPs to spontaneous HCCs *in situ*

*Mdr2* knockout mice develop spontaneous HCCs with age, mirroring disease progression in humans. Tumors are usually observed by 6–8 months of age, with males older than 10 months typically developing multiple large HCCs. The *Mdr2* knockout mouse is a physiologically relevant model for human HCC because, similar to humans with chronic liver disease, HCCs in these mice arise from malignant transformation of injured hepatocytes on a background of progressive fibrosis or cirrhosis.

**Figure 3:**
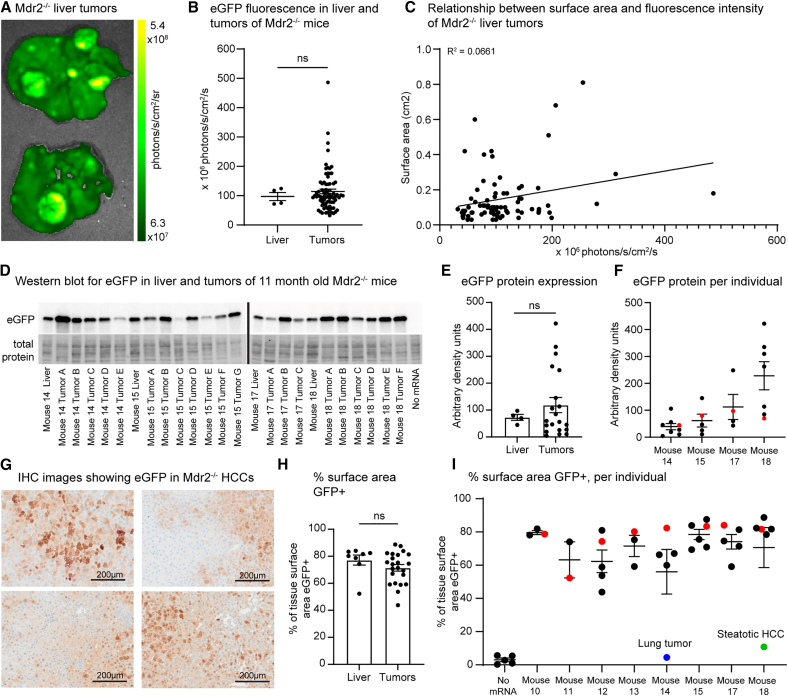
Delivery of mRNA-LNPs to spontaneous hepatocellular carcinomas *in situ* (A) Fluorescence image showing large HCCs on the livers of *Mdr2*^−/−^ mice. (B) Plot of fluorescence intensity of whole livers (*n* = 4) and individual tumors visible on the external surfaces of the same livers (*n* = 77). There was no significant difference in fluorescence intensity between livers and tumors (Mann-Whitney test, *n* = 4–77, U = 146, *p* = 0.8738). Error bars represent mean ± SEM. (C) There is no correlation between the external surface area of liver tumors and the fluorescence intensity (simple linear regression, F(1,75) = 5.308, *p* = 0.0240, R^2^ = 0.06609). (D) Western blotting detects eGFP from livers and tumors of 4 *Mdr2*^−/−^ mice 11 months of age. The quantity of eGFP protein detected from tumors is broadly comparable to that detected from livers and also shows variation. (E) Quantitation of eGFP protein expression determined by western blotting, showing greater variability in protein expression in tumors (*n* = 21) than in liver tissue (*n* = 4). The abundance of eGFP protein in tumors was not significantly different from liver tissue (Welch’s *t* test, *n* = 4–21, t = 1.519(21.99), *p* = 0.1430). Error bars represent mean ± SEM. (F) Quantitation of eGFP protein separated by animal; the red point in each column represents the eGFP abundance in liver tissue, and the black points represent the tumors from the same animal. Error bars represent mean ± SEM. (G) Representative IHC images show a range of possible outcomes for eGFP expression in tumor tissue, including broadly good delivery, variability in staining intensity between adjacent cells and between different regions of the image, and borders between stained and unstained regions of the tumor, which are otherwise similar in appearance. (H) Analysis of IHC images with *StainDetectAI* shows that the surface area of tissue sections with detectable IHC staining is not significantly different between liver tissue and tumors (Mann-Whitney test, *n* = 8–25, U = 60, *p* = 0.1580). Error bars represent mean ± SEM. (I) Quantification of IHC surface area positive for eGFP separated by animal; the red point in each column represents the stained surface area in liver tissue, and the black points represent the tumors from the same animal. Note poor delivery to a lung adenocarcinoma (blue; eGFP not detected above background level) and a steatotic HCC (green; eGFP detection marginal.) Figure partially created in BioRender. Leighton, L. (2026) https://BioRender.com/hxslnmy.

To investigate the efficacy of mRNA-LNP delivery in a physiologically relevant HCC model, we first compared eGFP expression between liver tissue and liver tumors of 11-month-old male *Mdr2*^−/−^ mice using *ex vivo* fluorescence imaging. Notably, many liver tumors were evident on the external surfaces of the livers (Figure S10), including several large tumors with increased fluorescence relative to the surrounding liver tissue (Figures 2B and 3A). The average fluorescence of the tumors (262.6 × 10^6^ photons/s/cm^2^/sr) was not significantly different from that of the liver tissue (97.1 × 10^6^ photons/s/cm^2^/sr) (Mann-Whitney test, *n* = 4–77, *p* = 0.8738) (Figures 3B and S11). There was no correlation between tumor size and fluorescence intensity (simple linear regression, R^2^ = 0.0661) (Figure 3C).

Using western blotting, we found that expression of eGFP protein in tumors was comparable to the expression in liver tissue, with considerable variability in protein abundance observed between individual tumors (arbitrary protein density units; tumors: range 4.3–774.9, average 150.1; liver tissue: range 44.9–97.1, average 72.9) (Figures 3D–3F). There was no significant difference between the average amount of eGFP protein detected in tumors and liver tissue (Welch’s *t* test, *n* = 4–21, *p* = 0.1430) (Figure 3E).

To investigate the pattern of eGFP expression at a cellular level, IHC was performed on sections of multiple individual tumors per mouse. IHC for eGFP in tumor sections confirmed that eGFP expression was variable both within tumors (with all tumors including cells both lightly and intensely stained for eGFP) and between tumors (with some tumors showing more stained cells and/or more intense staining than others) (Figures 3G and S12). We again used our custom model *StainDetectAI* to determine the percentage of tumor section surface area that exhibited positive GFP staining and found that surface area staining of spontaneous HCCs ranged from 43.7% to 88.7% (Figures 3H and 3I). There was no significant difference in the percentage of surface area stained between liver tissue and tumors (Mann-Whitney test, *n* = 8–25, *p* = 0.1580) (Figure 3H). Notably, tumor C from animal 18 was the only steatotic HCC in the dataset, and eGFP expression in this tumor was almost undetectable via both western blotting and IHC (Figures 3D–3I and S13). Additionally, a spontaneous adenocarcinoma of the lung was incidentally recovered from one of the 11-month-old animals. This tumor was also analyzed with *StainDetectAI*, and eGFP staining was not detected above background levels (Figures 3I and S14).

Given that angiogenesis is a feature of HCC and that changes to tissue vascularity are a feature of the *Mdr2* knockout mouse model,^42^ we investigated tissue vascularity by counting the number of blood vessels observed in 5 mm^2^ of tissue (Figure S15). There was no significant difference in blood vessel count between healthy liver and *Mdr2*^−/−^ liver or between *Mdr2*^−/−^ liver and *Mdr2*^−/−^ tumors (Dunn’s test, *n* = 8–23, *p* = 0.27). *Mdr2*^−/−^ tumors had slightly fewer blood vessels than healthy liver (Dunn’s test, *n* = 9–23, *p* = 0.0131). The modest reduction in blood vessel density observed in tumors relative to healthy liver could, in principle, reduce the opportunity for LNP extravasation into tumor tissue.

These findings demonstrate that intravenous delivery of mRNA-LNPs results in specific expression within most hepatocytes and many cells within spontaneously occurring HCCs *in situ*, with minimal delivery to other organs and tissues, likely including other cancers. This restricted delivery pattern suggests that mRNA-LNP therapeutics for HCC could be administered systemically with limited extrahepatic off-target activity.

### Delivery of mRNA-LNPs to a xenograft model of primary liver cancer

**Figure 4:**
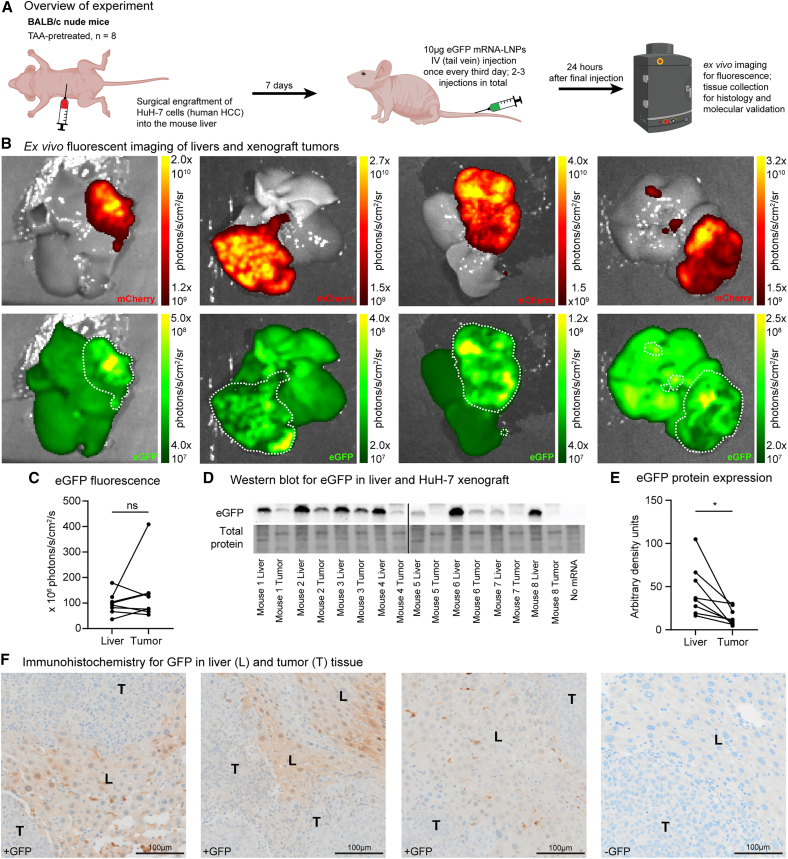
Delivery of mRNA-LNPs to a xenograft model of primary liver cancer (A) We surgically engrafted HuH-7 human HCC cells, which constitutively express mCherry, into the livers of BALB/c nude mice. Tissues were analyzed approximately 24 h after an injection of 10 μg eGFP mRNA-LNPs via the tail vein. (B) *Ex vivo* fluorescent imaging of entire livers with xenograft-derived tumors shows strong eGFP fluorescence in the liver tissue and also the tumors, marked by expression of mCherry. (C) Quantitation of eGFP fluorescence confirms broadly comparable signal between tumors and non-tumor liver tissue, with no significant difference found between liver and tumor (Wilcoxon matched-pairs signed-rank test, *n* = 8, W = 10.00, *p* = 0.5469). (D) Western blotting detects eGFP from all livers and tumors, with noticeably higher signal in the liver tissue and some bands from tumor detected very faintly. (E) Quantitation of eGFP protein expression determined by western blotting, showing lower signal in liver than tumor of each animal (paired *t* test, *n* = 8, t = 3.168(7), *p* = 0.0158). (F) Representative immunohistochemistry images of liver and tumor tissue from 3 different animals show clear GFP staining in HuH-7-derived tumors (T) and staining of a higher intensity in adjacent liver tissue (L). An image of HuH-7-derived tumor and adjacent liver tissue from a mouse not injected with eGFP and stained with anti-GFP antibody is provided for comparison. Figure partially created in BioRender. Leighton, L. (2026) https://BioRender.com/9ywif1v.

Animals bearing tumor xenografts derived from human cancer cell lines are a commonly used model system for testing new anti-cancer therapeutics. To determine whether mRNA-LNPs are delivered to liver tumor xenografts as in spontaneously occurring HCCs, we established an orthotopic xenograft model using HuH-7 human HCC cells modified to constitutively express mCherry and firefly luciferase (Figure S16). Cells were engrafted into the livers of thioacetamide (TAA)-treated BALB/c nude mice by single direct injection. Due to the aggressive growth of the HuH-7 tumors, mice were injected with eGFP mRNA-LNPs every third day beginning after a 1-week tumor establishment period, and when estimated tumor size reached 1cc, mice were euthanized 24 h after the most recent eGFP mRNA-LNP injection (Figure 4A). Mice received either 2 or 3 injections in total; as eGFP protein has a half-life of approximately 24 h,^43^ the detected signal predominantly reflects expression from the final dose.

To investigate the delivery of mRNA-LNPs to the tumors, we first used *ex vivo* fluorescent imaging of the eGFP reporter. Fluorescent signal well above background level was observed in the livers (range 36.4–179.1, average 97.9 × 10^6^ photons/s/cm^2^/sr) and liver tumors (range 54.6–408.7, average 135.4 × 10^6^ photons/s/cm^2^/sr) of all mice (*n* = 8) (Figures 4B and 4C). There was no significant difference in fluorescent signal between liver tissue and tumors (paired *t* test, *n* = 8, *p* = 0.3496). We considered the possibility that residual healthy liver tissue overlying the tumors might take up mRNA-LNPs and express eGFP, resulting in high tumor-surface fluorescence values that may not represent the uptake and expression of mRNA within the tumor interior. Therefore, to investigate penetration of mRNA-LNPs into the tumor interior, we performed fluorescence imaging on cross-sections of several tumors and observed mostly uniform fluorescence intensity within the tumors (Figure S17). We also expected that mRNA-LNP penetration might be better in small tumors than in large tumors, but we found that there was no correlation between tumor size (measured by area of the region of interest drawn around the tumor) and fluorescence intensity (simple linear regression, R^2^ = 0.0002) (Figure S18A). Surprisingly, there was also no correlation between the fluorescence intensity measured from the liver and tumor from the same animal (simple linear regression, R^2^ = 0.1620) (Figure S18B).

Western blotting supported the findings from fluorescence imaging: a clear eGFP band was detected in tumor samples, but with variability in band intensity (Figure 4D), with bands from two tumors detected extremely faintly. Protein expression was significantly lower in the tumor samples than in the liver tissue (range 16.3–104.9 and average 45.3 in the liver and range 4.7–30.0 and average 14.9 in the tumors) (paired *t* test, *n* = 8, *p* = 0.0158) (Figure 4E). IHC for eGFP in 3 tumors showed weak expression of eGFP in virtually all tumor cells, with some cells at the margins of most tumors showing higher signal (Figures 4F and S19). Expression of eGFP was substantially weaker in the HuH-7 tumors relative to the adjacent liver tissue. These results demonstrate that it is possible to deliver mRNA-LNPs to orthotopic xenograft models of primary liver cancer; however, the tumors showed less cell-to-cell variation in protein expression level and less overall protein expression relative to the spontaneously occurring HCCs of *Mdr2*^−/−^ mice.

Given the known role of LDLR in uptake of LNPs by healthy liver tissue, we performed IHC for LDLR on sections containing both liver and tumor tissue from 3 representative mice and found that, qualitatively, expression of LDLR was comparable between the liver and tumor tissue (Figure S20). We also considered the potential impact on tissue architecture, and specifically tumor cell density, on the extravasation of LNPs and subsequent reporter delivery after observing that the xenografts consisted of small, tightly packed cells. We manually counted nuclei from one 500 × 500 pixel field of view and compared the nuclei count (a proxy for cell density) between healthy liver, cirrhotic liver, tumors of *Mdr2*^−/−^ mice, and xenografted tumors (Figure S21A). Unlike either cirrhotic liver or spontaneous HCCs of the *Mdr2*^−/−^ mice, tumors derived from HuH-7 xenografts contained significantly more nuclei per unit area than healthy liver tissue (Dunnett’s T3 multiple comparisons test, *n* = 3, adjusted *p* = 0.0021), indicating dense packing of cells within the xenograft that is expected to have negatively impacted LNP extravasation and reporter expression. Qualitatively, the blood vessel density of HuH-7 xenograft tumors was comparable to that of healthy liver tissue (Figure S21B).

Notably, mCherry-positive foci were detected in the lungs of 3 animals from this cohort, indicating spontaneous metastasis of the xenografted HuH-7 cells from the liver to the lung. eGFP fluorescence was not detected in these small lung metastases above the background fluorescence level of normal lung tissue (Figure S22), suggesting that the location of the tumor in the liver is necessary for mRNA-LNP uptake and expression.

### Delivery of mRNA-LNPs to secondary liver tumors

**Figure 5:**
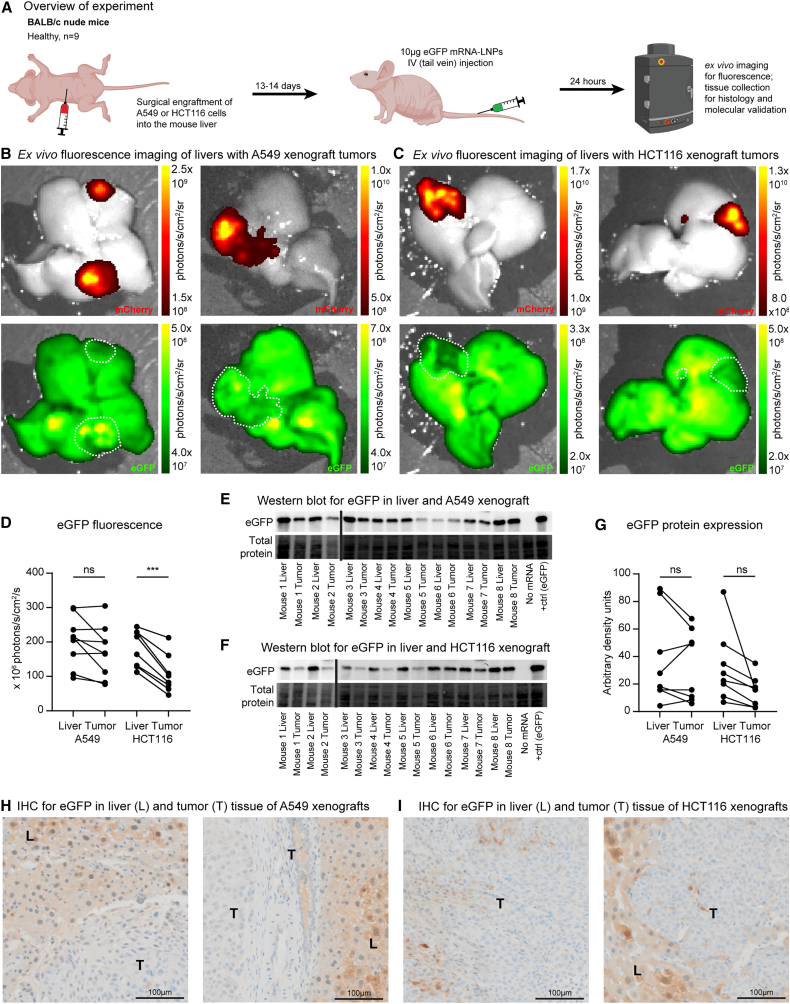
Delivery of mRNA-LNPs to secondary liver tumors (A) We surgically engrafted A549 (human lung adenocarcinoma) and HCT116 (human colorectal carcinoma) cells, which constitutively express mCherry, into the livers of BALB/c nude mice. Tissues were analyzed approximately 24 h after an injection of 10 μg eGFP mRNA-LNPs via the tail vein. (B and C) *Ex vivo* fluorescent imaging of entire livers with xenograft-derived tumors shows strong eGFP fluorescence in both liver tissue and tumors (marked by expression of mCherry). For A549-derived tumors (B), fluorescence was visually comparable, while for HCT116-derived tumors (C), fluorescent signal within the tumor was generally visibly lower than the adjacent liver tissue. (D) Quantitation of eGFP fluorescence for A549-derived tumors shows that fluorescence trends lower in tumors, but there is no significant difference (paired *t* test, *n* = 9, t = 2.269(8), *p* = 0.0530). In HCT116-derived tumors, fluorescence is significantly reduced relative to liver tissue (paired *t* test, *n* = 8, t = 7.293(7), *p* = 0.0002). (E) Western blotting detects eGFP from all livers and tumors of animals with A549-derived xenografts, with comparable or slightly lower band intensity in tumors relative to livers. (F) Likewise, western blotting detects eGFP from all livers and tumors of animals with HCT116-derived xenografts, with slightly lower band intensity for most tumors relative to livers. (G) Quantitation of eGFP protein expression determined by western blotting showed broadly comparable protein abundance in A549-derived tumors relative to liver tissue (paired *t* test, *n* = 8, t = 0.7250(7), *p* = 0.4920). In HCT116-derived tumors, the abundance of eGFP protein trended lower but was not significantly different from liver tissue (paired *t* test, *n* = 8, t = 2.240(7), *p* = 0.0601). (H) Immunohistochemistry images of liver and tumor tissue from two different animals with A549-derived tumors, showing faint staining for eGFP in tumor cells (T) and strong signal in adjacent liver (L). (I) Immunohistochemistry images for liver and tumor tissue from two animals with HCT116-derived tumors, showing faint to absent staining for eGFP in tumor cells (T) with strong signal in adjacent liver (L). Figure partially created in BioRender. Leighton, L. (2026) https://BioRender.com/dln0uyn.

It is currently unknown whether cancer cells derived from other tissues can take up mRNA when engrafted in the liver. To model secondary liver cancer, we established derivatives of two human cancer cell lines, HCT116 (colorectal carcinoma) and A549 (lung adenocarcinoma), which constitutively express mCherry (Figure S16). These cell lines were chosen because cancers of the colon and lung are among the most common primary sources for secondary liver tumors. Cells were engrafted into the livers of BALB/c nude mice by single direct injection (*n* = 8–9), and after approximately 2 weeks, mice were injected with 10 μg of eGFP mRNA-LNPs via the tail vein and then culled 24 h later for tissue collection and imaging (Figure 5A).

Using *ex vivo* fluorescence imaging, we again observed eGFP fluorescence well above background in liver tissue of all mice. eGFP fluorescence was also detected in all tumors derived from both A549 and HCT116 (Figures 5B and 5C). We again used fluorescence imaging of tumor cross-sections to confirm mRNA expression within tumor interiors (Figure S23). In A549 xenografts, fluorescence was comparable between liver (range 95.6–299.2 × 10^6^ photons/s/cm^2^/sr, average 203.4 × 10^6^ photons/s/cm^2^/sr) and tumor (range 77.1–304.9 × 10^6^ photons/s/cm^2^/sr, average 172.0 × 10^6^ photons/s/cm^2^/sr) (paired *t* test, *n* = 9, *p* = 0.0530) (Figures 5B–5D). However, in HCT116 xenografts, fluorescence was significantly lower in the tumors (range 45.9–212.4 × 10^6^ photons/s/cm^2^/sr, average 106.2 × 10^6^ photons/s/cm^2^/sr) than in the liver tissue (range 112.1–244.5 × 10^6^ photons/s/cm^2^/sr, average 181.6 × 10^6^ photons/s/cm^2^/sr) (paired *t* test, *n* = 8, *p* = 0.0002) (Figures 5C and 5D). For both A549 and HCT116 xenografts, there was no correlation between tumor size and fluorescence intensity (Figures S24A and S24C), but there was a moderate to strong correlation between the fluorescence intensity measured for the liver tissue and tumor from the same animal (Figures S24B and S24D).

Western blotting confirmed the presence of eGFP protein in all tumors, again with considerable variability in band intensity (Figures 5E and 5F). There was no significant difference in eGFP protein abundance between liver tissue and tumors in the A549 group (range 4.2–89.3 and average 37.6 in liver, range 5.9–67.5 and average 33.6 in tumors) (paired *t* test, *n* = 8, *p* = 0.4920) (Figure 5G). Similarly, in the HCT116 group, there was no significant difference in eGFP abundance between liver and tumors, although the abundance in tumors trended lower (range 6.8–86.9 and average 32.3 in liver, range 2.9–35.1 and average 14.9 in tumors) (paired *t* test, *n* = 8, *p* = 0.0601) (Figure 5G).

IHC for eGFP in two A549 tumors (Figures 5H and S25A) and two HCT116 tumors (Figures 5I and S25B) showed weak expression of eGFP in most tumor cells and much stronger eGFP expression in adjacent healthy liver. These results indicate that mRNA-LNPs can be delivered to hepatic xenografts of non-liver cancer types.

Expression of LDLR in A549 and HCT116 tumors was examined by IHC from 3 representative mice per cell line. We found that, qualitatively, expression of LDLR was higher in A549 tumors than in healthy liver tissue and comparable between HCT116 tumors and liver tissue, with some hyperintense cells observed in the xenograft (Figure S20). We also investigated the cell density and tissue vascularity of xenograft-derived secondary liver tumors (Figure S21A). Significantly increased cell density was observed in xenografts derived from both A549 cells (Dunnett’s T3 multiple comparisons test, *n* = 3, adjusted *p* = 0.0011) and HCT116 cells (Dunnett’s T3 multiple comparisons test, *n* = 3, adjusted *p* < 0.0001). Qualitatively, the blood vessel density in both xenografts appeared lower than in healthy liver tissue (Figure S21B). Both factors may have impeded LNP extravasation and may partially explain weaker expression of eGFP in these tumors.

As with the HCC xenografts, these tumors show lower levels of eGFP protein expression relative to the liver tissue and less cell-to-cell heterogeneity than was observed for spontaneous HCCs. mRNA-LNP uptake within secondary liver tumors substantially increases the potential scope of mRNA therapeutics for cancer.

## Discussion

In this study, we demonstrate the successful delivery of systemically administered mRNA-LNPs to the diseased and cancerous mouse liver. We observed mRNA expression in healthy, fibrotic, and cirrhotic mouse liver tissue; spontaneous HCC *in situ*; and xenograft models of HCC and secondary liver cancer. Using a custom machine learning approach, we quantified the ubiquitous distribution of mRNA expression in hepatocytes in healthy liver, while showing that in the presence of fibrosis, cirrhosis, and cancer, reporter expression varies both within and between samples. These findings demonstrate that intravenous administration of mRNA-LNP therapeutics can be a viable therapeutic strategy for liver disease and cancer and suggest future directions for research to improve delivery of mRNA drugs.

Many nanoparticle-based carrier systems have been developed for the delivery of mRNA *in vivo*, with mRNA-LNPs being the most widely used. mRNA-LNPs are manufactured using four lipid components: an ionizable lipid, a phospholipid, cholesterol, and a PEG-lipid, each of which can be substituted to alter the properties of the nanoparticle.^44^^,^^45^ The ionizable lipid is the most consequential component, influencing mRNA encapsulation efficiency, endosomal escape, tissue tropism, and immunostimulatory profile.^44^^,^^45^ A small number of ionizable lipids are used in RNA-LNP products that have received regulatory approval for clinical use (including DLin-MC3-DMA, ALC-0315, and SM-102), with others currently in clinical development (notably including SM-86, used for a hepatocyte-targeted gene replacement therapy currently in clinical trials^33^). In this study, we used SM-102, which is among the most extensively characterized ionizable lipids in the preclinical mRNA literature, facilitating direct comparison with existing and future studies. Importantly, while DLin-MC3-DMA was developed for targeted delivery of small interfering RNAs (siRNAs) to hepatocytes, SM-102 outperformed it for expression of an mRNA reporter gene.^46^^,^^47^ It should be noted that delivery efficiency, biodistribution, and immunostimulatory profile can vary across ionizable lipid formulations,^38^ and the findings of this study may not generalize to other LNP formulations; evaluation of alternative formulations represents a valuable direction for future work. Additionally, we tested a single dosage of mRNA-LNPs in this study, using a reporter gene only; future studies should also evaluate the effect of mRNA-LNP dosage on the distribution and intensity of transgene expression across disease models, as the relative expression between liver and tumor cells may be dose dependent.

The eGFP expression measured in this study reflects the combined efficiency of LNP accumulation, cellular uptake, endosomal escape, and mRNA translation, and the relative contribution of each step may differ between healthy tissue, fibrotic liver, and tumors. Previous studies have tracked the biodistribution and intracellular fate of mRNA-LNPs using fluorescent labeling of both LNPs and mRNA cargo^48^ and have characterized the pharmacokinetics and tissue distribution of mRNA-LNPs formulated with SM-102 and other clinically relevant ionizable lipids.^46^ In the present study, we measured protein expression as the functional endpoint most relevant to therapeutic applications, as it integrates all upstream delivery steps into a single readout of productive transfection.

When mRNA-LNPs are injected intravenously, they rapidly adsorb a protein corona that prominently includes the serum lipid transport protein ApoE,^49^ which can act as a ligand for the low-density lipoprotein receptor (LDLR). This receptor is expressed on the surface of many cell types and is particularly abundant on hepatocytes, which are the primary site for low-density lipoprotein (LDL) clearance from the blood.^50^ Interaction of ApoE-coated LNPs with LDLR is one mechanism promoting LNP uptake by hepatocytes; however, the relative importance of this mechanism depends on the LNP formulation, and LNPs remain hepatotropic in LDLR knockout mice.^51^ The protein corona of mRNA-LNPs is complex, and in addition to ApoE, also includes albumin, other apolipoproteins, and complement factors; its composition is also influenced by the LNP formulation and route of administration.^52^ Corona composition affects cellular tropism and the pathways by which LNPs are internalized, trafficked, and processed by cells.^53^

LNP uptake by hepatocytes is also facilitated by the fenestrated epithelium of the liver, which allows selective permeability of large particles into liver tissue, which would be excluded from entry into other tissue types by the endothelial barrier.^50^ Accumulation of nanoparticle drug carriers, particularly lipid-based carriers, in the liver is a well-documented phenomenon,^26^^,^^50^^,^^54^ and many prior publications have shown that systemically administered mRNA-LNPs are predominantly and abundantly expressed in the healthy liver, specifically by hepatocytes.^27^^,^^28^^,^^29^ Delivery of mRNA to hepatocytes is a viable treatment option for several diseases, including genetic metabolic disorders where protein replacement within hepatocytes, or secretion of the protein from hepatocytes into the bloodstream, is adequate to treat the disease. A notable example is mRNA-3927, a drug in clinical trials for treatment of propionic acidemia.^33^

To date, few studies involving mRNA-LNP delivery to the liver have considered the impact of hepatic fibrosis or cirrhosis. Liver fibrosis, characterized by the deposition of bands of extracellular matrix proteins within the liver tissue, results from chronic liver injury from a range of causes, including chronic viral hepatitis, alcohol abuse, drug or chemical exposures, MASLD, and several genetic conditions. The end stage of this process is cirrhosis, characterized by extensive replacement of liver tissue with scar tissue so that liver function is significantly impaired.^55^ In these disease states, the increased intracellular matrix within the space of Disse and capillarization of the sinusoids impedes blood flow and the transfer of molecules between sinusoids and hepatocytes. Additionally, increased interstitial fluid pressure in fibrotic and cirrhotic liver tissue impedes nanoparticle extravasation and diffusion into the tissue.^56^ The applicability of mRNA therapeutics targeting the liver in the presence of chronic liver disease is thus partly dependent on the efficacy of mRNA-LNP delivery in the presence of these ultrastructural and morphological changes. One recent study described generally good mRNA-LNP delivery to the liver in mouse models of fibrosis and cirrhosis.^34^ In this study, we build on these findings by using multiple orthogonal methods to describe the range of outcomes possible for mRNA-LNP delivery in the presence of severe chronic liver disease.

To investigate the impact of severe liver fibrosis or cirrhosis on mRNA-LNP delivery, we used the *Mdr2* knockout mouse, which develops progressive fibrosis and cirrhosis due to bile leakage.^39^^,^^40^ Imaging of whole livers from *Mdr2*^−/−^ mice injected with eGFP mRNA-LNPs revealed significant eGFP fluorescence in the liver tissue, with both hypointense and hyperintense tumors evident. eGFP IHC performed on liver tissue sections from these animals showed considerable heterogeneity both within and between samples. Scattered hepatocytes with strong eGFP expression were a feature of virtually all sections, although their density varied considerably. Notably, we observed marginal to undetectable eGFP expression within fibrotic tissue tracts, consist mainly of extracellular matrix and fibroblasts rather than hepatocytes. Our finding that the surface area of tissue sections with any reporter delivery was negatively correlated with the severity of fibrosis suggests that severe liver damage does impair delivery of mRNA-LNPs; however, moderately effective delivery was achieved even in mice with cirrhosis. Therefore, severe liver disease is unlikely to be a contraindication to the usage of mRNA drugs targeting the liver, although the dosage or frequency of administration may need to be adjusted to ensure adequate delivery in this patient population. Furthermore, mRNA therapies show promise for the direct treatment of liver injury and chronic liver disease. *Hnf4a* mRNA inhibits the development of liver fibrosis in several animal models,^34^ and expression of growth factors using mRNA-LNPs can reverse liver pathology and support engraftment of healthy hepatocytes to treat liver disease.^29^^,^^57^

Our study also systematically investigated mRNA-LNP delivery to liver cancer. *Mdr2*^−/−^ mice develop HCCs with age, and we therefore examined reporter expression in spontaneous HCCs *in situ*. While a small number of reports previously described the use of mRNA-LNP-based therapies to treat liver cancer in animal models,^58^^,^^59^^,^^60^^,^^61^ the encoded therapeutic proteins act through non-cell-autonomous mechanisms of action, and delivery of mRNA-LNPs to tumor cells was unclear. We found that GFP was expressed in HCCs with a similar distribution and intensity to its expression in the liver tissue from the same animal. Cellular heterogeneity is a common feature of HCCs,^62^ and this may explain our observation that several HCCs contained tracts of cells that did not express GFP. It would be valuable for future work to explore sequential delivery of multiple reporter mRNA-LNPs in order to determine whether repeated dosing of mRNA-LNPs would target the same or different cell populations. Interestingly, we found one steatotic HCC among the *Mdr2*^−/−^ tumors, and GFP expression was virtually absent from this tumor. However, robust GFP expression was observed in small steatotic regions within liver and tumor sections from other animals in this cohort. This finding warrants further investigation to determine whether steatotic livers or steatotic HCCs are especially poor targets for mRNA-LNP delivery.

As an alternative model for HCC, we also examined mRNA delivery in mice bearing xenografts of the human HCC cell line HuH-7. Fluorescence imaging showed comparable expression of eGFP in the livers and tumors of these animals. However, western blotting showed reduced eGFP expression in tumors relative to adjacent liver tissue, including two tumors from which the eGFP band was barely detectable. IHC also showed much stronger eGFP staining in adjacent liver tissue than in tumors. We also considered the delivery of mRNA-LNPs to mice bearing xenografts of two human cell lines of non-liver origin, as a model for secondary liver cancer. Reporter expression was demonstrated in all tumors derived from both cell lines using multiple orthogonal methods, but expression was weak in comparison to the adjacent liver tissue.

Interestingly, delivery of mRNA-LNPs into the naturally occurring HCCs of the *Mdr2* knockout mice was more efficient than delivery into any of the xenograft tumors. This is likely due to the difference in cell size and density; *Mdr2*^−/−^ tumors comprised much larger cells than xenografts derived from any of the three cell lines we considered. Studies of tissue penetration have found that nanoparticles larger than 20 nm struggle to penetrate tumors due to their high cell density and interstitial fluid pressure.^63^^,^^64^^,^^65^ High cell density and high tumor rigidity are typical of animal models bearing tumor xenografts derived from human cell lines, and the weaker delivery of mRNA-LNPs to these tumor models may represent an inherent limitation of the model system rather than the delivery technology. In contrast, the *Mdr2* knockout mouse model develops progressive liver disease that progresses through the stages of fibrosis, cirrhosis, and spontaneous development of HCCs; this is a physiologically relevant model that recapitulates the disease process most commonly leading to HCCs in humans. Therefore, better performance of mRNA-LNPs in this model compared to xenograft models is a positive indicator of their applicability for cancer therapy. Our study also informs the choice of animal model for future research into mRNA-LNP therapeutics for liver cancer. The relatively weak delivery of mRNA-LNPs to orthotopic xenografts suggests that this model may underestimate the efficacy of mRNA cancer therapies and that a mouse model that develops HCCs spontaneously is more likely to respond to treatment. Future studies could also consider syngeneic models of primary liver cancer (for instance, hepatic engraftment of Hepa1-6 tumors in C57/L mice) and secondary liver cancer (such as MC38 or CT26 implanted by intrasplenic injection in C57BL/6 or BALB/c, respectively); like most spontaneous cancer models, syngeneic models would allow the study of mRNA-LNP delivery and expression in the context of a complete immune response, addressing an inherent limitation of the athymic xenograft models used in this study.

Nanoparticle composition for targeted mRNA delivery is an area of active research.^38^^,^^44^^,^^45^ For instance, mRNA-LNPs with substituted lipid components enable selective targeting of the liver, lung, and spleen^28^; other publications have described nanoparticle formulations that are optimized for RNA delivery to different types of liver cells^66^ or for co-delivery of siRNA, single-guide RNA (sgRNA), and mRNA in a single LNP.^58^ Excitingly, several technologies are in development that may improve the ratio of protein expression in tumor cells relative to liver cells, enabling therapeutic benefit with reduced mRNA-LNP dosage and reduced side effects related to expression of the mRNA in healthy hepatocytes. Cell-selective targeting including to extra-hepatic tumors can be achieved using bispecific antibodies that recognize a tumor cell-surface marker and a component of the LNP,^66^^,^^67^ and other studies have shown enhanced mRNA penetration into tumors when co-delivered with a therapeutic siRNA to reduce tumor rigidity,^58^ as well as improved specificity of mRNA for tumors over hepatocytes by including binding sites for hepatocyte-specific miRNAs in the mRNA design.^68^ It is also important to note that many emerging RNA-based treatments for solid tumors do not require direct transfection of tumor cells. For instance, most cancer vaccines are administered by the subcutaneous or intramuscular route and rely on transfection of antigen-presenting cells to produce an adaptive immune response against the tumor.^69^^,^^70^ Other therapeutic approaches target stromal cells, endothelial cells, or tumor-associated macrophages, and these approaches also do not require transfection of the tumor cells themselves^69^; however, improved targeting of mRNA therapeutics to tumor-associated cell types will enhance the efficacy of these emerging therapeutic modalities. Another area of active research is delivery of other nucleic acid modalities such as siRNAs and antisense oligonucleotides, which differ substantially in size, structure, and pharmacology from the mRNA used in this study and are often delivered using different nanoparticle formulations.^69^

Another noteworthy finding of our study is the immune response, including PD-L1 elevation, in the liver 24 h after injection of mRNA-LNPs. Exogenous mRNA is immunostimulatory through interactions with Toll-like receptors and cytosolic RNA sensors, and components of the mRNA-LNP (notably, the SM-102 ionizable lipid used in this study) are also immunostimulatory.^38^ Our findings are consistent with previous reports of immune activation after mRNA-LNP administration. Importantly, one study reported that the inflammatory response in the liver was attenuated by reducing the dosage of mRNA-LNPs.^34^ This highlights the importance of ongoing improvements to the design of mRNA medicines to enable reduction in mRNA dosage while still achieving therapeutic levels of protein expression.

In conclusion, we investigated reporter expression after systemic administration of mRNA-LNPs by the intravenous route in mice. Building on previous findings showing strong expression of mRNA in hepatocytes, we have demonstrated mRNA expression in the healthy liver, fibrosis, cirrhosis, spontaneous HCCs, and xenograft models of both primary and secondary liver cancer. Our results show that systemically administered mRNA-LNPs can transfect cells across multiple models of liver disease and cancer, supporting the potential of this drug modality for the treatment of liver cancer.

## Materials and methods

### Ethics statement

This study complies with the Australian Code for the Care and Use of Animals for Scientific Purposes. All procedures involving live animals were approved by the Institutional Animal Care and Use Committee of The University of Queensland (ethics approval certificates 2021/000492 and 2023/000234).

### Animals

Male BALB/cOzarc mice were obtained from Ozgene (Perth, Australia).

Male BALB/c nude (BALB/c-*Foxn1*^*nu*^/Ozarc) mice were obtained from Ozgene (Perth, Australia).

Male *Mdr2*^−/−^ (FVB.129P2-*Abcb4*^*tm1Bor*^/J) mice were obtained from a breeding colony maintained by K.R.B. and X.L. at the Pharmacy Australia Center of Excellence (Brisbane, Australia).

For imaging controls only, surplus or ex-breeder mice (male BALB/c and male and female CD1) were obtained from a training colony maintained at the Australian Institute for Bioengineering and Nanotechnology (Brisbane, Australia).

BALB/c and BALB/c nude mice used for baseline delivery and xenograft experiments were 8–10 weeks old when experiments commenced. *Mdr2*^−/−^ mice were 2–11 months old as indicated. BALB/c and CD1 imaging controls were 3–9 months old.

All animals used in this study were group housed (3–5 per cage) on a 12-h light-dark cycle at ambient temperature, with *ad libitum* access to food and water and with enrichment items (cotton nesting material, shredded cardboard, cardboard domes, and popsicle sticks) provided in cages.

### Cells

HuH-7 human HCC cells were purchased from ATCC. HCT116 human colorectal carcinoma cells were a kind gift from Professor Michael McGuckin’s group. These cell lines were maintained in DMEM (Gibco 11965-092) supplemented with 10% fetal bovine serum (FBS) (Gibco 10100147) and 1% penicillin/streptomycin (1,000 U/mL, Gibco 15140122).

A549 human lung adenocarcinoma cells were a kind gift from the laboratory of Professor Helmut Schaider. A549 cells were maintained in RPMI 1640 medium (Sigma-Aldrich R8578) supplemented with 10% FBS, 1% pen/strep, 2 mM L-glutamine (Thermo Fisher Scientific 25030081), 1 mM sodium pyruvate (Thermo Fisher Scientific, 11360070), and 15 mM HEPES (Thermo Fisher Scientific, 15630080).

All cells were maintained at 37°C with 5% CO_2_ in a humidified incubator. The cell lines used in this study are not listed in the International Cell Line Authentication Committee and National Center for Biotechnology Information Biosample database of misidentified cell lines. All cell lines used in this study tested negative for mycoplasma.

To generate derivatives of A549 and HCT116 that constitutively express mCherry, cells were seeded into 6-well plates and then co-transfected with the pBRPB CAG-mCherry-IP plasmid (Addgene #106333) and the piggyBac transposase plasmid (PBase) at a molar ratio of 3:1 using Lipofectamine 3000 (Invitrogen L3000001) according to the manufacturer’s instructions. After 48 h, puromycin selection was applied (6 mg/mL for A549 and 5 mg/mL for HCT116) and maintained for 2 weeks. To generate a derivative of HuH-7 that constitutively expresses mCherry and firefly luciferase, cells were seeded into 6-well plates and then co-transfected with the pB-EF1a-FLuc-IRES-Puro plasmid and PBase at a 3:1 molar ratio using Lipofectamine 3000. After 48 h, cells were selected with puromycin (5 mg/mL) for 2 weeks. This cell line was then co-transfected with the pBRPB CAG-mCherry-IP plasmid and PBase at a 3:1 molar ratio and selected with puromycin (5 mg/mL) for a further 2 weeks. The BD FACSAria Fusion instrument was used to select cells with red fluorescence, and this cell population was retained. All knock-in cell lines are routinely maintained in nonselective media.

To prepare cells for xenografting, cultures were grown to between 50% and 90% confluent and not passaged within the 24 h prior to harvest. Cells were trypsinized and washed twice with PBS and then pelleted by gentle centrifugation and resuspended in undiluted Matrigel (Corning FAL354277). Cell suspensions were maintained on ice and used within 5 h of preparation.

### Production of mRNA-LNP

mRNA-LNPs were produced according to a method published previously.^71^^,^^72^ Briefly, a synthetic gene encoding eGFP was obtained from a custom DNA synthesis provider (Integrated DNA Technologies, Singapore). DNA was amplified using 2X Q5 PCR master mix (New England Biolabs M0494L) with a forward primer containing a T7 promoter and a reverse primer encoding a 126-nt poly(A) tail. The PCR product was purified and used as a template for *in vitro* transcription of mRNA using T7 RNA polymerase (New England Biolabs M0251L), with 100% substitution of uridine residues by N1-methylpseudouridine (BOC Sciences 1429803-59-6.) The synthesized mRNA was purified, integrity verified by capillary electrophoresis, and function verified by transfection into mammalian cell culture. mRNA-LNPs were produced with the following lipid molar ratios: 50 ionizable (SM-102)/10 DSPC/38.5 cholesterol/1.5 DMG-PEG2000. Formulation was conducted on the Nanoassemblr Ignite with an N/P ratio of 6.8, a total flow rate of 12 mL/min, and an aqueous:organic flow rate ratio of 3:1. Following formulation, mRNA-LNPs were buffer-exchanged into Tris-buffered saline (TBS) by dialysis and concentrated by centrifugation on a size-exclusion column. A sample of concentrated mRNA-LNPs underwent quality control assessment using the Zetasizer to confirm that size, charge, and polydispersity were within the expected range (Figure S26). Remaining concentrated mRNA-LNPs were adjusted to a final sucrose concentration of 10% (w/v) as a cryoprotectant and stored at −30°C in single-use aliquots at an mRNA concentration of 700–1,100 ng/μL.

### mRNA delivery in healthy BALB/c mice

Male BALB/c mice 16 weeks old were injected via tail vein with 10 μg of mRNA-LNPs diluted to a final volume of 100 μL with TBS. Approximately 24 h after injection, mice were euthanized and organs removed for imaging.

### mRNA delivery in *Mdr2*^−/−^ mice

4 groups of male *Mdr2*^−/−^ mice (*n* = 4–5) were defined based on age: 2–3 months, 5 months, 8 months, and 11 months. Mice were injected via tail vein with 10 μg of mRNA-LNPs diluted to a final volume of 100 μL with TBS. Approximately 24 h after injection, mice were euthanized, and their organs were collected for histology and molecular analysis. A separate cohort of 11-month-old *Mdr2*^−/−^ males (*n* = 4) was used for imaging; these mice were injected via tail vein with 10 μg of mRNA-LNPs diluted to 100 μL with TBS, then euthanized approximately 14 h later, and their organs were removed for fluorescence imaging.

### mRNA delivery in an orthotopic xenograft model of human liver cancer

Male BALB/c nude mice (*n* = 9) were provided with drinking water containing 200 mg/L thioacetamide (TAA) for 8 weeks. 4–5 days after discontinuation of TAA treatment, mice underwent surgery to engraft human cancer cells (HuH-7-FLuc-mCherry.) Briefly, mice were anesthetized with isoflurane and then injected subcutaneously with 0.1 mg/kg buprenorphine for analgesia, and ophthalmic lubricant was applied. Mice were gently secured in dorsal recumbency using paper tape, and the surgical site was prepared with 3 sequential povidone-iodine scrubs. A 2 cm midline abdominal incision was made using scissors and the liver was partially exteriorized onto sterile gauze swabs drenched in normal saline. 1 × 10^6^ cells suspended in 20 μL of undiluted Matrigel (Corning FAL354277) were injected into the left lateral lobe of the liver using a 29G insulin syringe. Hemostasis was achieved using gentle pressure with cotton swabs wet with normal saline, then the abdomen was closed in 2 layers using 6/0 braided silk sutures (Ethicon 15232-ET) in a simple interrupted pattern. Subcutaneous buprenorphine 0.1 mg/kg was provided twice daily for 3 days for postoperative pain relief.

Beginning 7 days postoperatively, mice underwent twice weekly bioluminescent imaging for monitoring of tumor size. Briefly, mice were injected subcutaneously with 100 mg/kg D-luciferin (Vivo-Glo, Promega 1042) and then imaged 15 min later using the IVIS Lumina X5 under light isoflurane anesthesia. mRNA-LNPs encoding eGFP (10 μg in 100 μL of TBS) were injected via tail vein on each imaging day. Mice were euthanized 24 h after mRNA-LNP injection when the estimated tumor size reached 1 cc or on day 15 post-xenograft. The 8 animals included in data analysis were therefore euthanized on day 12 (*n* = 1) or day 15 (*n* = 7). One animal required euthanasia for welfare criteria at day 7 (prior to mRNA injection) and was therefore excluded from the study.

### mRNA delivery in xenograft models of secondary liver cancer

Male BALB/c nude mice were used for this experiment. No TAA treatment was performed. Mice underwent surgery for cancer cell engraftment according to the procedure described for the HuH-7 xenograft experiment. Mice received either 1 × 10^6^ A549-mCherry cells (*n* = 9) or 2 × 10^6^ HCT116-mCherry cells (*n* = 9). 13–14 days postoperatively, mRNA-LNPs encoding eGFP (10 μg in 100 μL of TBS) were injected via tail vein. Mice were euthanized approximately 24 h later, and their organs were removed for fluorescence imaging. One mouse in the HCT116 group required euthanasia for welfare criteria prior to mRNA injection and was therefore excluded from the study.

### Fluorescent imaging of mouse organs

Following euthanasia, organs were removed onto chilled metal trays covered with opaque black plastic. Fluorescent imaging was performed using the IVIS Lumina X5 operated using LivingImage 4.7.4 (PerkinElmer). Images were acquired using the following settings: field of view B, fluorescence filter pair software presets for mCherry and eGFP, automatic exposure, and cosmic ray correction on.

For image display, the RedHot lookup table was used and intensity-to-color scales were set with reference to uninjected controls to best capture the qualitative data contained within the images. For images representing eGFP fluorescence, a custom script was used to invert the R and G color values of exported PNG images to create a “GreenHot” image.

Data analysis was performed using LivingImage 4.7.4 (PerkinElmer). To obtain numerical data for fluorescence of organs and tissues, a region of interest (ROI) was drawn around each tissue or organ and the fluorescence (average radiant efficiency) was recorded. Background fluorescent signal (arising from tissue autofluorescence) was estimated for each organ by averaging the fluorescent signal obtained from that organ from 2 to 6 uninjected mice. For mice injected with eGFP mRNA-LNPs, eGFP fluorescence for each organ was determined by subtracting the average background fluorescence from the same organ.

### Tissue processing and IHC

Tissue samples used for histology and IHC were drop-fixed by placing them in 10% neutral buffered formalin (Sigma-Aldrich F5554-4L) as soon as practical after dissection and imaging. After 24 h of fixation, tissues were transferred to 70% ethanol for short-term storage and then paraffin embedded using a standard 9-h processing cycle. 4 μm paraffin sections on plain glass slides were used for H&E and picrosirius red stains. 4 μm paraffin sections on Uberfrost+ slides were used for IHC. Prior to IHC, antigen retrieval was performed using standard protocols (EDTA at pH 9 for GFP, and citrate at pH 6 for LDLR). IHC was performed using the Ventana Discovery Ultra automated stainer (Roche Diagnostics). For detection of GFP, rabbit anti-GFP primary antibody (Novus NB600-308) was used at 1:1,000. For detection of LDLR, rabbit anti-LDLR primary antibody (Thermo 10785-1-AP) was used at 1:500. Bright-field images were captured using the Olympus VS120 slide scanning microscope at 20× (H&E stain) or 40× (IHC) magnification.

### IHC image analysis with a custom AI model, *StainDetectAI*

IHC images were analyzed using *StainDetectAI*, which was developed to enable automated analysis of the entire IHC image dataset rather than a subsample. Existing software often uses techniques such as pixel thresholding, color deconvolution, and rule-based morphology. These approaches typically require manual interventions such as parameter tuning and are poorly suited to scalable, high-throughput analysis. To overcome these limitations, *StainDetectAI* was developed as a fully automated image segmentation solution tailored to the requirements of this study. *StainDetectAI* is a convolutional neural network (CNN) machine learning model prepared using standard U-Net architecture^73^ with four encoding and decoding layers. Each layer included convolutional blocks with ReLU activation functions and batch normalization. The model was implemented using the PyTorch 2.4 library^74^ and trained to distinguish stained and unstained areas of mouse liver and spontaneous HCC using annotated tiles extracted from GFP IHC slide images from the *Mdr2* KO dataset. “No staining” was defined based on GFP IHC on liver sections from mice not injected with mRNA (Figures S3 and S4). Training was performed using the Adam optimizer,^75^ a learning rate of 0.0001, the mean Intersection over Union (mIoU) loss function, and early stopping after 30 epochs of no improvement. The final model finished training with a cell classification accuracy of 94.4% and precision of 97.7%, and its predictions were manually validated.

To determine the stained surface area of mouse liver and tumor sections, microscopy images in .vsi format were extracted using a custom script and split into 512 × 512 pixel tiles. Tiles were analyzed by the trained model, and a prediction mask was generated. The number of pixels in each tile corresponding to a prediction of stained, unstained, and “other” (e.g., empty sections of the slide) was then summed and recorded for each sample. The entire area of each section was considered, and where available, two or more consecutive sections from the same sample were averaged together to calculate the final staining percentage.

For the subsample analysis reported in Figure S8, microscopy images in .vsi format were extracted using a custom script and split into 512 × 512 pixel tiles. Tiles were manually inspected, and empty tiles, “edge tiles” (containing 10% or more of the slide background at the edge of the tissue section), and tiles containing mostly fibrotic tissue or damaged regions of the section were deleted. For each liver, 10 of the remaining tiles were randomly selected, and regions of the image containing fibrotic bands were manually masked. *StainDetectAI* was run on the unmodified tile images, and then data corresponding to the masked regions were removed to calculate the stained surface area of the remaining parts of the tiles.

### Other histological analyses

For counting of nuclei as a proxy for cell density, virtual slide images were displayed at 10× on a high-definition computer monitor and a screen capture taken. A 500 × 500 pixel tile was created from an area of the section that contained only typical cells for the tissue type being considered. (Tiles were captured away from section edges, blood vessels, bile ducts, fibrotic bands, etc.) Nuclei within the tile were manually counted to estimate cell density.

For counting of blood vessels, the total number of blood vessels within 5 mm^2^ of tissue was estimated by counting the number of vessels present in each of 5 fields of view at 20× magnification.

### Protein extraction and western blotting

Tissue samples used for western blotting were snap-frozen on dry ice as soon as practical after dissection and imaging. Frozen tissue samples (3–30 mg) were disrupted in 200–300 μL cold RIPA lysis buffer (Cell Signaling Technologies 9806) supplemented with HALT protease and phosphatase inhibitor cocktail (Thermo Fisher Scientific 78440), using a glass Dounce homogenizer, then vortexed thoroughly and incubated on ice. Homogenates were centrifuged for 10 min at 10,000 × *g* and 4°C, and then the supernatant was transferred to a new tube, vortexed, and aliquoted for storage at −80°C. Protein samples were quantified by BCA assay using the Pierce reducing agent compatible microplate BCA protein assay kit according to the manufacturer’s directions (Thermo Fisher Scientific 23252). SDS-PAGE was performed using 10 μg of total protein per lane on Stainfree AnyKD mini TGX precast gels (Bio-Rad 4568126). Following activation of the Stainfree reagent using the ChemiDoc MP (Bio-Rad), protein was transferred onto low-fluorescence polyvinylidene fluoride (PVDF) membranes (Bio-Rad) using the Trans-Blot Turbo (Bio-Rad) with the “1X Mini-TGX” preset (2.5 A and 25 V for 3 min). Membranes were blocked in EveryBlot blocking buffer (Bio-Rad 12010020) and then incubated overnight at 4°C with primary antibodies diluted in blocking buffer. Membranes were then washed and probed using fluorescent secondary antibodies diluted in a mixture of 50% TBS (150 mM NaCl, 50 mM Tris-HCl, pH 7.6) and 50% EveryBlot blocking buffer with 0.02% SDS. Antibody details are listed in Table S2. Blots were imaged using the ChemiDoc MP. Quantitative image analysis was performed using Image Lab 6.1 software, and protein levels were normalized to total protein signal detected using the Stainfree reagent.

### RNA extraction, RT-qPCR, and RNA sequencing

Tissue samples used for RNA extraction were snap-frozen on dry ice as soon as practical after dissection and imaging. Frozen tissue samples (3–30 mg) were disrupted in 500 μL room temperature Nucleozol (Macherey-Nagel 740404.200) using a glass Dounce homogenizer. Water was added and samples were centrifuged to remove contaminants according to the manufacturer’s directions. Supernatant was then mixed 1:1 with ethanol, and RNA was captured on a Zymo-Spin IC column (Zymo Research R1013). DNase treatment was performed on-column, and RNA was washed and eluted according to the manufacturer’s directions. RNA integrity was verified using the Agilent 4200 TapeStation, and samples used for sequencing were RIN 8 and above. Stranded sequencing libraries were prepared from the poly(A) fraction of the RNA samples using the VAHTS Universal V8 RNA-seq library prep kit for Illumina according to the manufacturer’s directions. Libraries were sequenced using the Illumina Novaseq 6000 platform as 2 × 150 bp paired-end reads, at a depth of approximately 25 million reads per sample. Reads were aligned to the mouse genome (GRCm39) using STAR,^76^ and gene counts were obtained via featureCounts.^77^ Read counts were analyzed in Degust^78^ for differential gene expression using Voom/Limma, applying a threshold of ≥10 counts per million in at least three samples, false discovery rate (FDR) <1E−5, and fold change >2. Gene set enrichment analysis was performed using STRING.^79^

### Statistical analysis

Statistical analysis throughout was performed using GraphPad Prism 10.4.1. Where applicable, all statistical tests used in this study were two sided.

Prior to two-group comparisons, data were tested for normality using the Shapiro-Wilk test. For unpaired comparisons, normally distributed data were analyzed using Student’s *t* test (when standard deviations were similar) or Welch’s *t* test (when standard deviations differed.) Data that were non-normally distributed were analyzed using the Mann-Whitney test. For paired data, normally distributed data were analyzed using paired *t* tests, and the Wilcoxon matched-pairs signed-rank test was used otherwise.

To assess potential associations between continuous variables, simple linear regression analyses were performed based on the expectation of a proportional relationship. For each analysis, residuals were inspected to verify the assumptions of linearity and homoscedasticity.

## Data and code availability

The mouse liver RNA-seq dataset generated during this study is available through the NCBI Gene Expression Omnibus (GEO): GSE331154 (“Bulk RNA sequencing from liver of mice injected with saline or mRNA-LNPs encoding eGFP, 10 μg dose, 24 h time point, intravenous route.”) All other data supporting the findings of this study are available from the corresponding author upon reasonable request.

Code and documentation created during this study are available on GitHub: https://github.com/SidHow/StainDetectAI.

## Acknowledgments

We thank Dr. Haotian Yang and Dr. James Humphries for their generous advice and expertise. We acknowledge the facilities and scientific and technical assistance of the Center for Advanced Imaging at the Australian Institute for Bioengineering and Nanotechnology. We gratefully acknowledge advice and technical services from the Histology Facility and Microscopy Facility at the Translational Research Institute and UQ Biological Resources facilities. We acknowledge the facilities and the scientific and technical assistance of the BASE mRNA Facility (basefacility.org.au) and the National Biologics Facility (NBF; www.nationalbiologicsfacility.com) at The University of Queensland. BASE and NBF are supported by Therapeutic Innovation Australia (TIA). TIA is supported by the Australian Government through the National Collaborative Research Infrastructure Strategy (NCRIS) program. We acknowledge the following sources of funding and support: National Health and Medical Research Council (GNT2014002 and GNT1161832) to T.R.M., Australian Research Council (DE230100036 and FT250100341) to S.W.C., Medical Research Future Fund (MRFCRI000063 and MRFCRI000089) to S.W.C. and T.R.M., National Collaborative Research Infrastructure Strategy (NCRIS), Therapeutic Innovation Australia (TIA) to T.R.M. and S.W.C., Tour de Cure to S.W.C., D.H.G.C., L.J.L., and T.R.M., and The University of Queensland to S.W.C. and T.R.M.

## Author contributions

L.J.L. and S.W.C. conceived the study. L.J.L., K.R.B., X.L., and S.W.C. designed the experiments. L.J.L. performed animal surgeries and imaging. Y.J.G. produced and validated modified cell lines. L.J.L., S.U.M., M.V., Y.J.G., N.L.C., C.L.D.M., D.K.W., and K.R.B. performed tissue collection, sample processing, and molecular experiments. G.C.M. interpreted histology results. S.A.H. designed and wrote software used for image analysis. X.L. and K.R.B. manage the *Mdr2* knockout mouse colony and provided mice for the study. S.W.C., T.R.M., D.A.M., and D.H.G.C. provided resources and supervised the study. L.J.L. and S.W.C. wrote the manuscript. All authors reviewed the manuscript.

## Declaration of interests

T.R.M. and S.W.C. have received research funding from Oxford Nanopore Technologies, Sartorius Stedim Australia, and Sanofi. L.J.L., T.R.M., and S.W.C. have received support for conference attendance, travel, and accommodation from Moderna. No commercial entity was involved in this research.

## Supplemental information

Document S1. Figures S1–S26 and Table S2

Table S1. Genes differentially regulated in liver tissue 24 h after mRNA-LNP injection

Document S2. Article plus supplemental information
