# Supplementary material for: Systemically delivered mRNA-LNPs transfect primary and secondary liver tumors
Source: Mol Ther Nucleic Acids. 2026 Jun 17;37(3):102989. doi: 10.1016/j.omtn.2026.102989 (PMC13334401; doi:10.1016/j.omtn.2026.102989)
Supplement: Document S1. Figures S1–S26 and Table S2 [file mmc1.pdf]

OMTN, Volume *37*

Supplemental information

Systemically delivered mRNA-LNPs transfect primary and secondary liver tumors

Laura J. Leighton, Yee Jing Gee, Sachithrani U. Madugalle, Maria Victorova, Nissa L. Carrodus, Kim R. Bridle, Sidney A. Howell, Xiaowen Liang, Gregory C. Miller, Chris L. D. McMillan, Danushka K. Wijesundara, David A. Muller, Darrell H.G. Crawford, Timothy R. Mercer, and Seth W. Cheetham

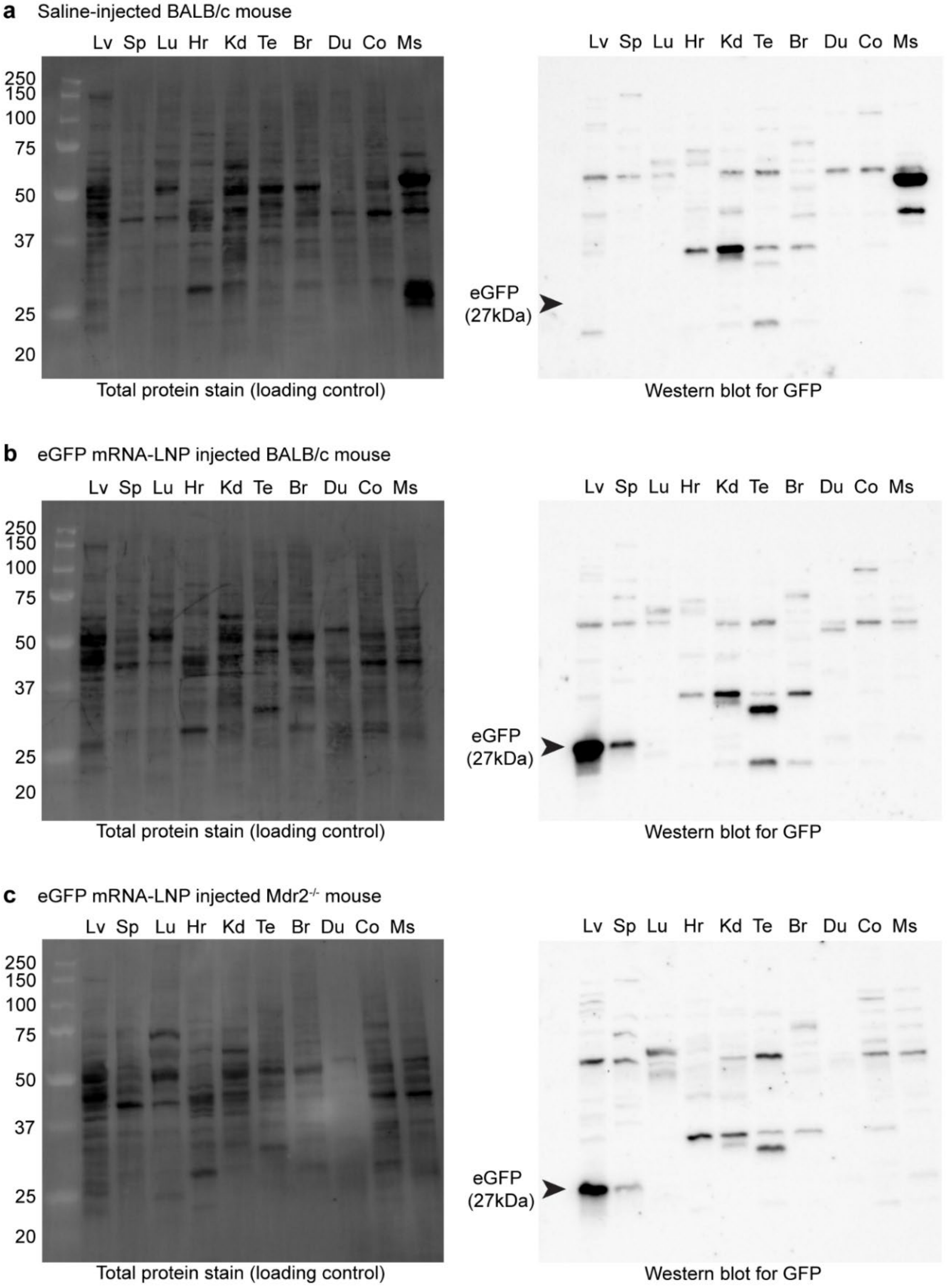

Figure S1: Biodistribution of eGFP protein (Western blot)

Western blotting was performed to detect eGFP from various organs and tissues of one mouse per group. Tissue was collected 24 hours after mRNA-LNP injection. **(a)** Saline-injected BALB/c mouse. **(b)** eGFP mRNA-LNP injected BALB/c mouse. **(c)** eGFP mRNA-LNP injected Mdr2$^{-/-}$ mouse. Lv=liver, Sp=spleen, Lu=lung, Hr=heart, Kd=kidney, Te=testis, Br=brain, Du=duodenum, Co=colon, Ms=mesentery. Bands corresponding to GFP are not observed in tissue lysates from the uninjected mouse. In both eGFP mRNA-LNP injected animals, a strong GFP band is detected from the liver and a moderate band from the spleen, with trace detection from some other organs.

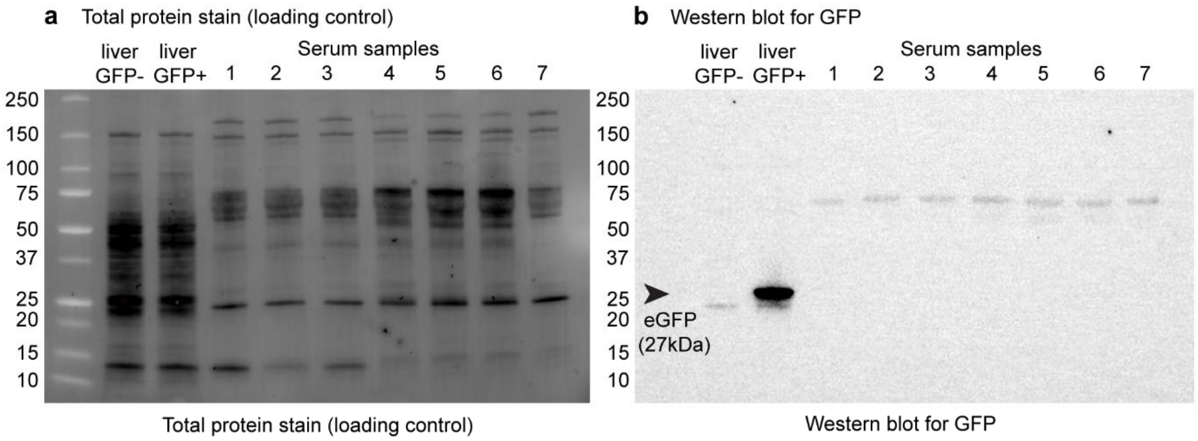

Figure S2: eGFP is undetectable in serum (Western blot)

Western blotting was performed to detect eGFP from serum, which was collected by cardiac puncture 24 hours after mRNA-LNP injection. Liver lysates from a saline-injected and eGFP mRNA-LNP injected mouse were run on the blot as negative and positive controls for eGFP detection. Serum from 3 healthy BALB/c mice injected with eGFP mRNA-LNPs (serum samples 1-3), 3 Mdr2$^{-/-}$ mice injected with eGFP mRNA-LNPs (serum samples 4-6), and one healthy BALB/c mouse injected with saline (serum sample 7) showed no detectable band corresponding to eGFP.

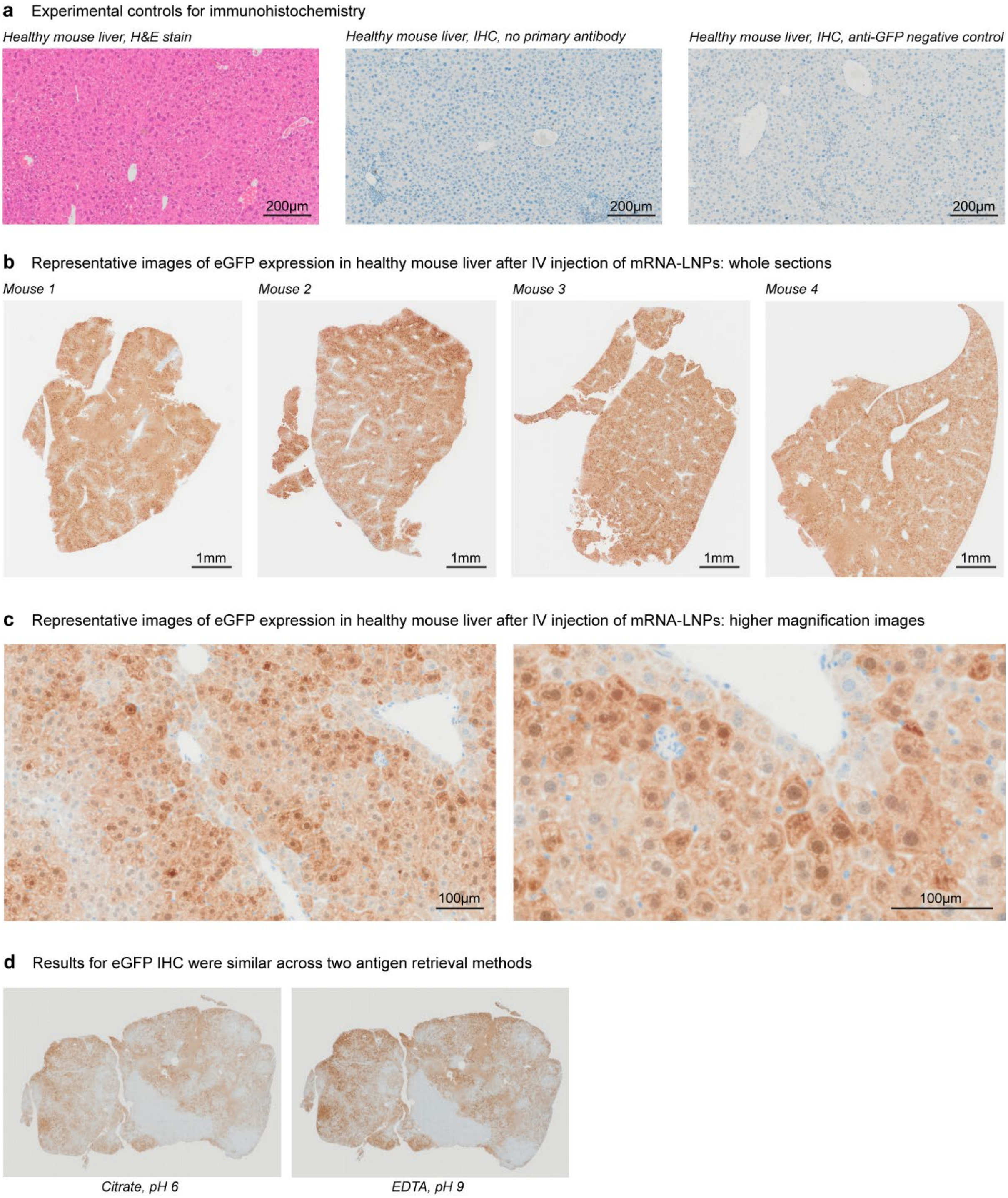

Figure S3: Additional histology images for healthy mouse liver

(a) H&E stain demonstrates normal morphology of healthy mouse liver. Immunohistochemistry controls demonstrate very low background of the assay. **(b)** whole section images taken from large pieces of mouse liver tissue (approx. 5 cubic mm) demonstrate that expression of eGFP from IV-injected mRNA-LNPs is strong and even throughout the liver tissue. **(c)** higher-magnification images demonstrate moderate to strong eGFP expression in all hepatocytes, with some expression visible in some endothelial cells. **(d)** There is a slight difference in staining intensity, and no difference in staining pattern, between two tested methods of antigen retrieval for GFP IHC; the EDTA, pH 9 method was selected and is used for all GFP IHC images in this study.

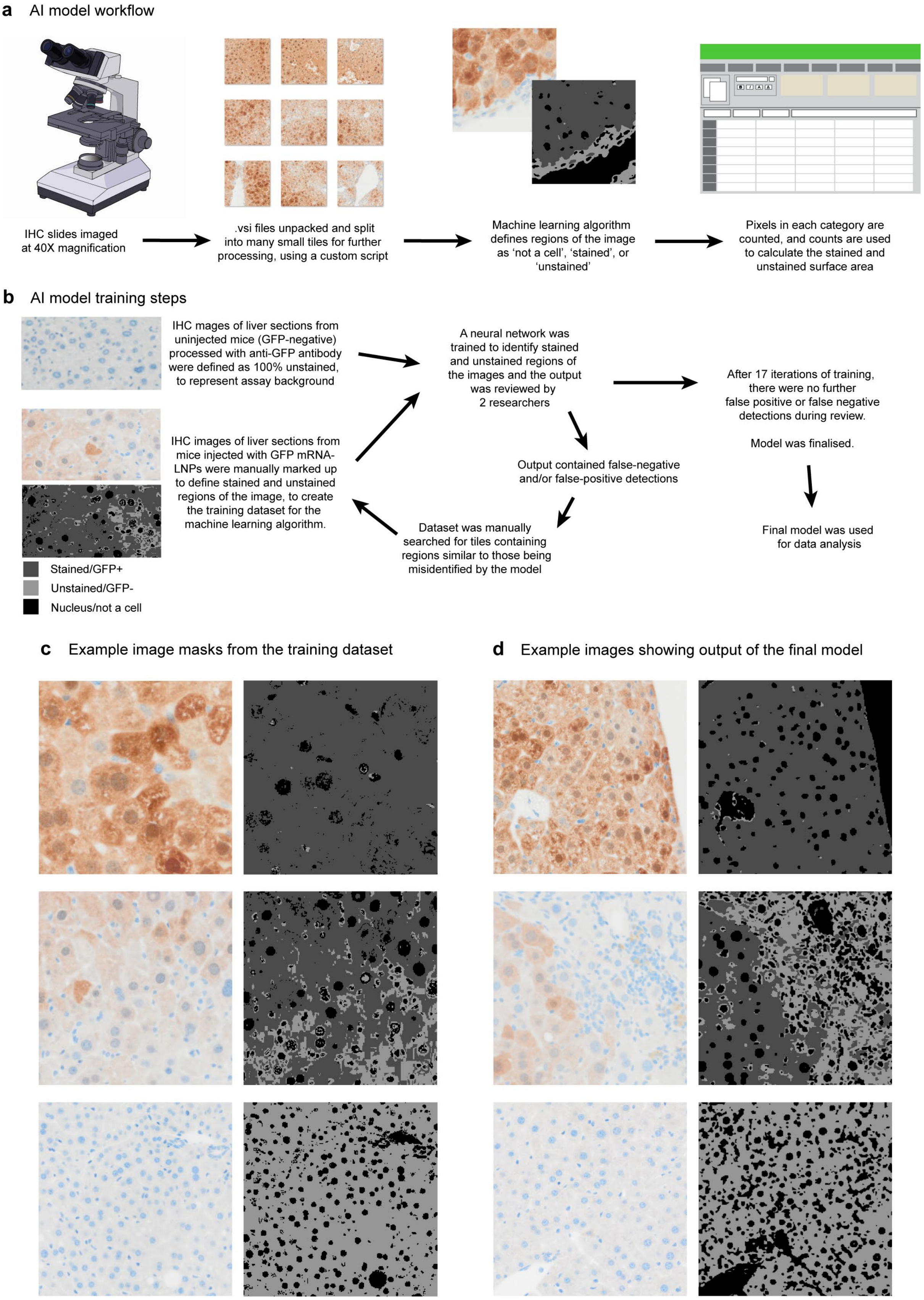

Figure S4: Overview of AI model used for IHC data analysis

(a) Flow chart of workflow for IHC data analysis using the custom AI model. **(b)** Flow chart of steps used to train the AI model to distinguish GFP-stained from unstained liver tissue. **(c)** Example image masks from the training dataset, used to provide input to the model. **(d)** Example output images from the final model, demonstrating its ability to accurately identify even light IHC staining with negligible false-positive detection. Partially created in BioRender. Leighton, L. (2026) https://BioRender.com/3f6jg4n

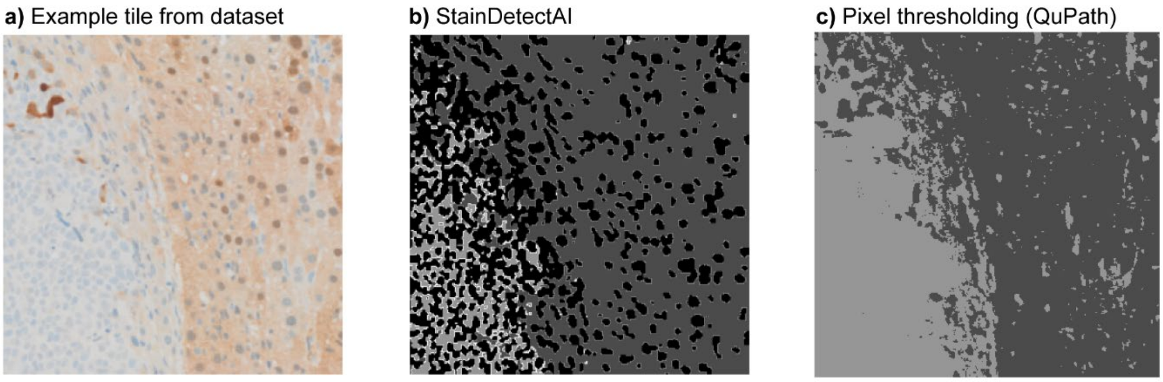

Figure S5: Comparison of StainDetectAI and pixel thresholding

(a) Example image taken from the IHC dataset generated for this project. **(b)** Output of the StainDetectAI model. Black areas correspond to nuclei, dark grey to DAB-HE stain, and light grey to unstained regions of the sample. 57.4% of pixels were classified as stained. **(c)** Output of DAB-HE pixel thresholding using the open-source bioimage analysis suite QuPath[1]. Dark grey areas correspond to DAB-HE stain, and light grey to unstained regions. 62.2% of pixels were classified as stained. This result shows that StainDetectAI and the QuPath pixel thresholder provide similar results, the difference attributable to nuclei being correctly identified by StainDetectAI.

[1]: Bankhead, P. *et al.* QuPath: Open source software for digital pathology image analysis. *Scientific Reports* (2017). https://doi.org/10.1038/s41598-017-17204-5

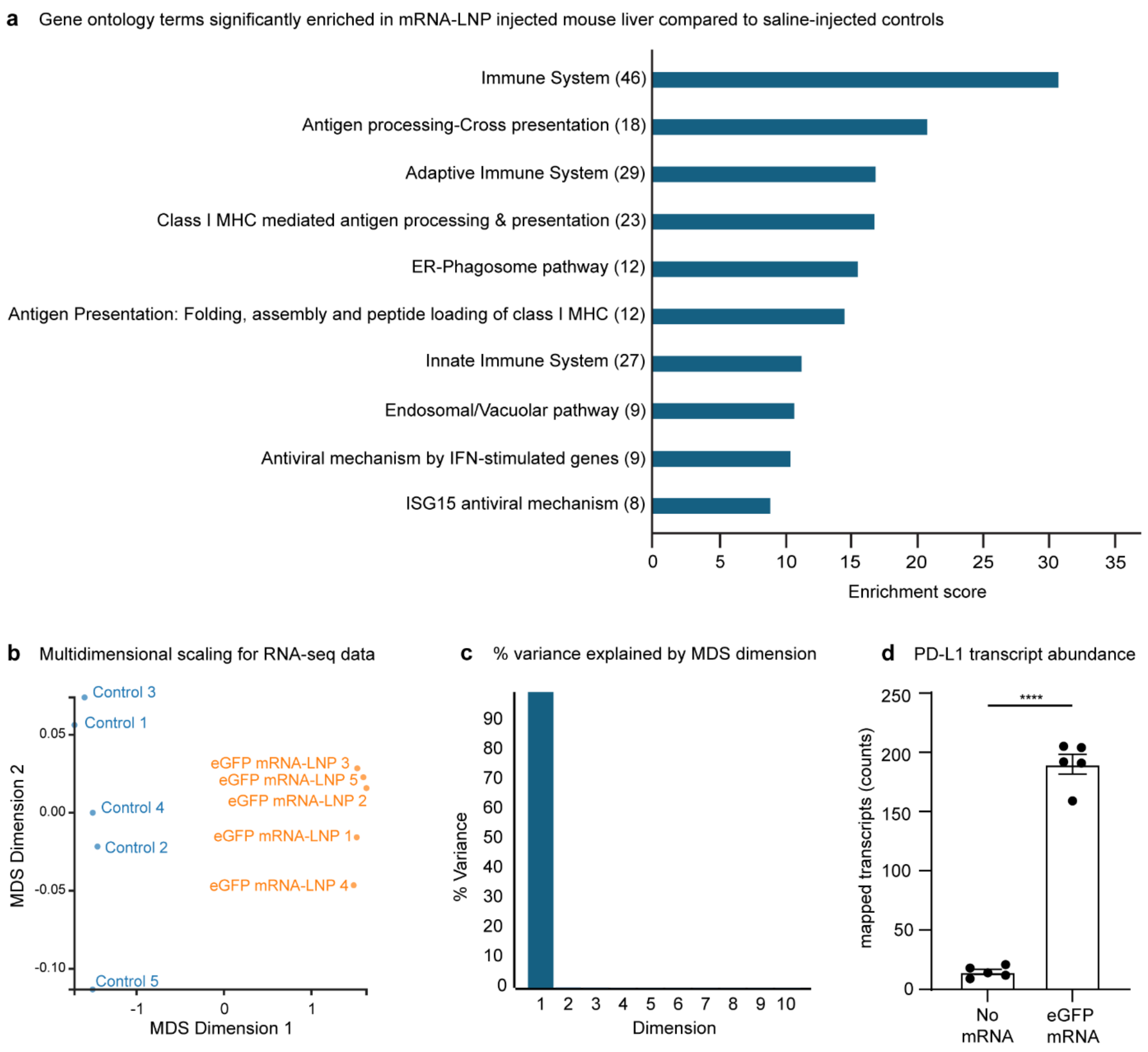

Figure S6: Additional results from RNA sequencing

(a) Gene ontology analysis of RNA sequencing data from the liver tissue of healthy mice injected with eGFP mRNA-LNPs compared to saline-injected controls. Enriched GO terms are related to the immune system and antigen processing and presentation. **(b)** Multidimensional scaling plot shows clear separation of mRNA-LNP injected animals from controls along MDS dimension 1. **(c)** MDS dimension 1 explains well over 90% of the variance in the data. **(d)** Abundance of CD274 (PD-L1) transcript was significantly increased in the mouse liver 24 hours after injection of eGFP mRNA-LNPs (Welch's t-test, n=5, t=20.41(4.522), $p<0.0001$).

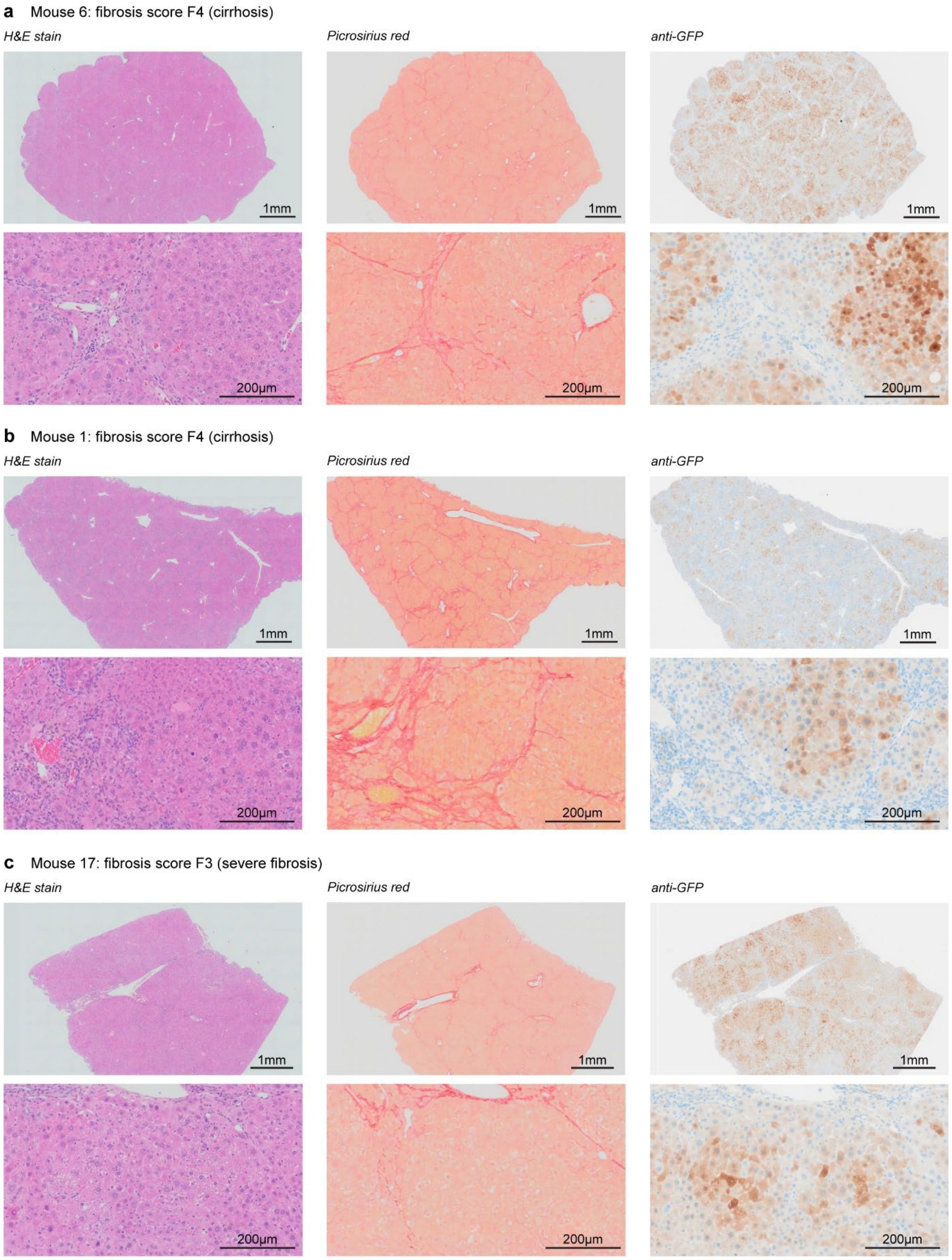

Figure S7: Additional histology images, Mdr2$^{-/-}$ liver tissue

Mdr2$^{-/-}$ mice develop bile leakage, severe fibrosis and cirrhosis. These images show the range of outcomes for liver disease severity and effectiveness of mRNA delivery. H&E stain (left) shows general tissue architecture, picrosirius red (centre) highlights collagen present in areas of fibrosis, and IHC for GFP (right) demonstrates delivery of mRNA-LNPs. **(a)** Typical delivery in an animal with cirrhosis (F4); 75% of the liver section surface area is positive for GFP. **(b)** Poor delivery in an animal with cirrhosis (F4); 44% of the liver surface area is GFP positive. **(c)** Typical delivery in an animal with severe fibrosis (F3); 84% of the liver surface area is GFP positive.

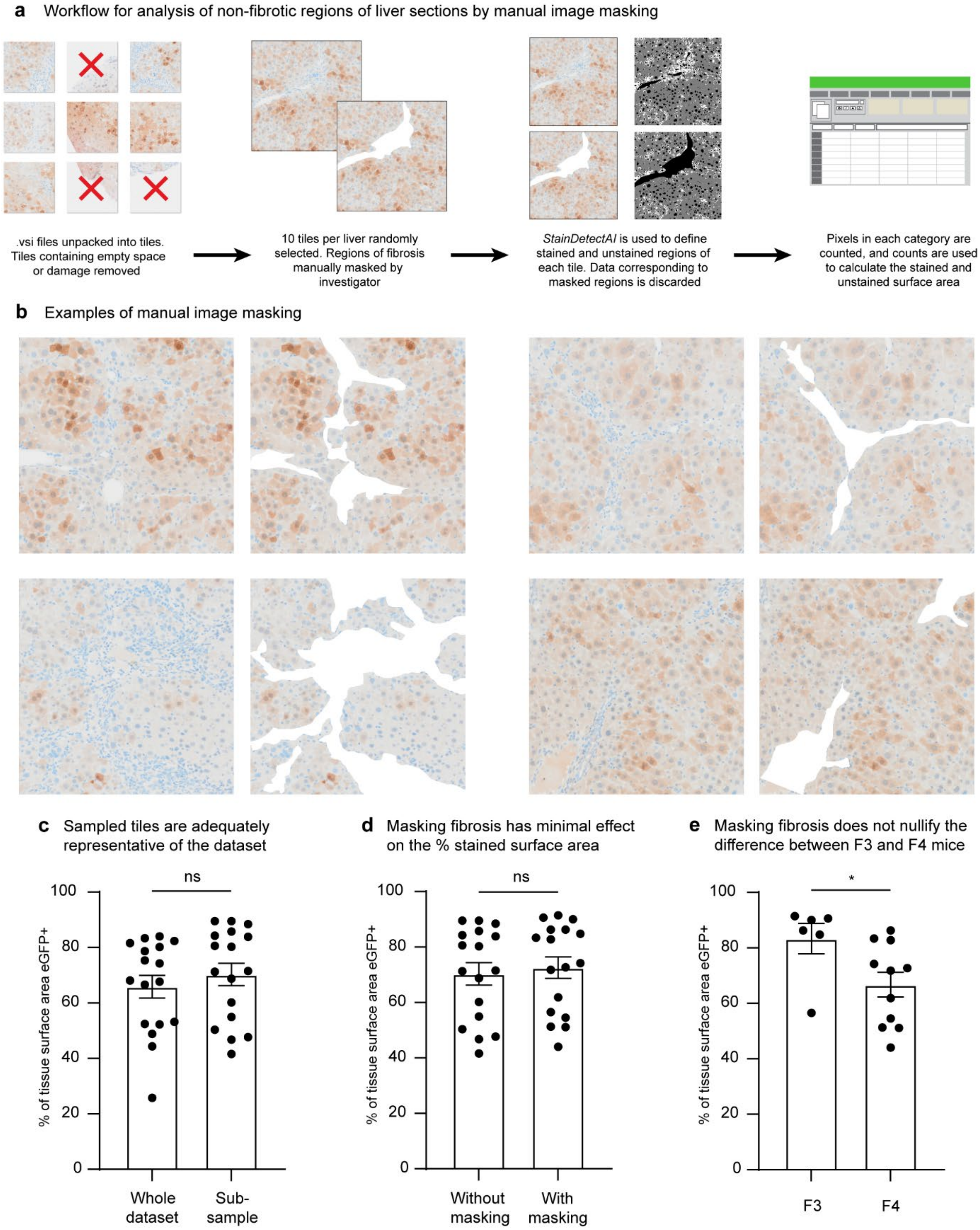

Figure S8: Analysis of IHC data subset with manual masking of fibrosis

(a) Flow chart of workflow for analysis of a random sample of IHC data from Mdr2$^{-/-}$ mice with manual masking applied to the images, to consider the eGFP expression distribution in only non-fibrotic regions of the tissue sections. **(b)** Example of IHC image tiles without (left) and with (right) the masking applied. **(c)** Comparison of stained surface area (measured using *StainDetectAI*) for the random dataset sample in comparison with the whole dataset indicates that the random sample is representative of the dataset as a whole (Mann-Whitney test, n=17, U=115, p=0.3180). **(d)** Comparison of stained surface area for the random dataset sample with and without masking shows that there was no significant difference in the percentage of staining identified after masking fibrotic regions (Mann-Whitney test, n=17, U=124, p=0.4901). **(e)** Consistent with the findings of the whole-dataset analysis, in the masked sample there is a significant reduction in stained surface area for liver sections from animals with a fibrosis score of F4, relative to those scoring F3 (Mann-Whitney test, n=17, U=8, p=0.0103).

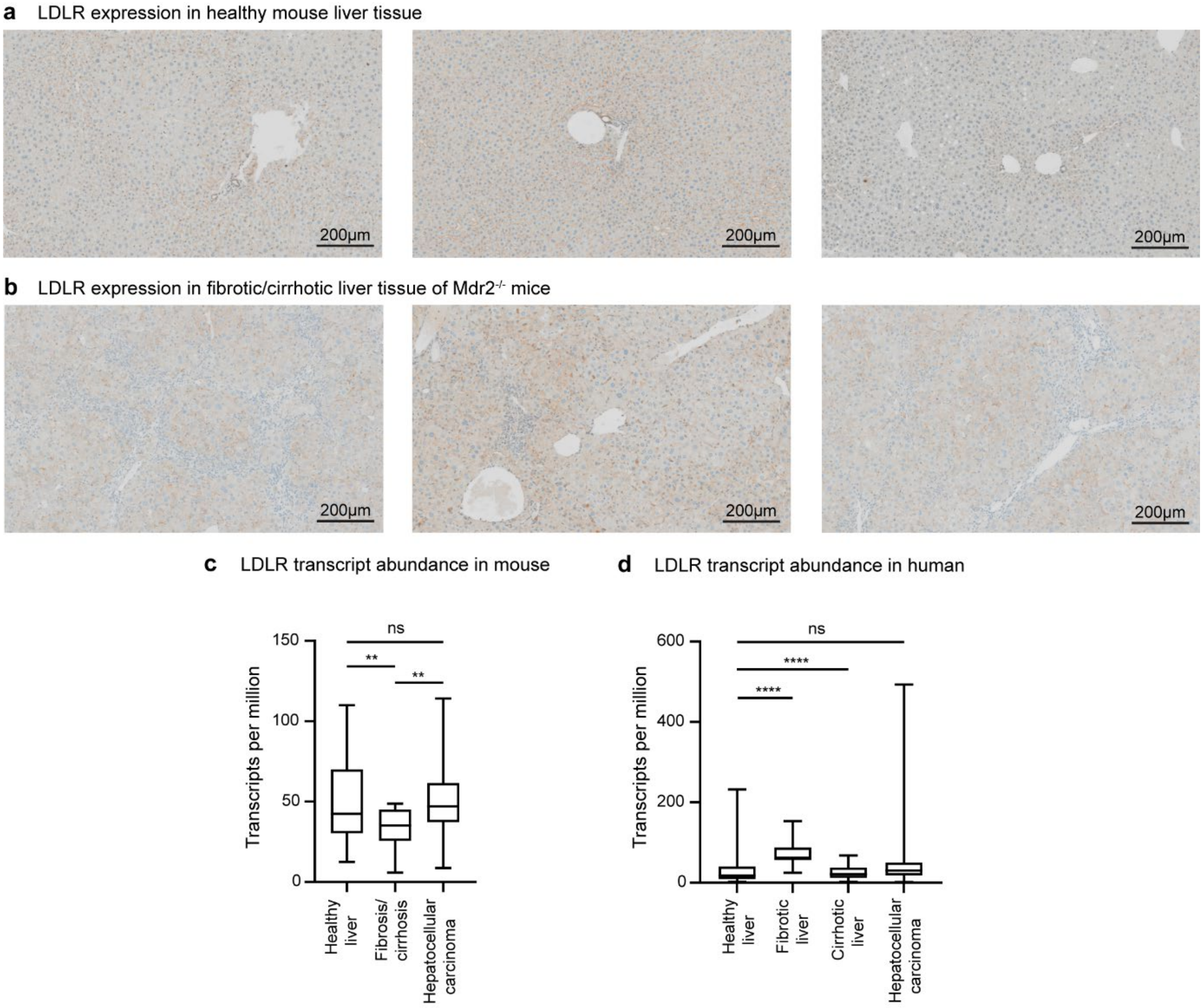

Figure S9: LDLR expression in liver fibrosis and cirrhosis

(a) Moderate to strong membrane-enhanced expression of the low-density lipoprotein receptor (LDLR) is observed in healthy liver tissue from 3 BALB/c mice. **(b)** In Mdr2$^{-/-}$ mice, LDLR staining is absent from fibrotic bands, but the staining pattern and intensity is otherwise similar to the healthy livers. **(c)** In composite mouse data from the GepLiver database, LDLR expression is significantly downregulated relative to healthy liver and HCC, which do not differ significantly from each other. (Kruskal–Wallis test with Dunn's post-hoc, overall difference among groups (H = 12.92, p = 0.0016). Significant pairwise differences: Healthy liver and Fibrotic liver (p=0.0041) and Fibrotic liver and HCC (p=0.0013); Healthy liver vs HCC was not significant. **(d)** In composite human data from the GepLiver database, LDLR expression is significantly upregulated in fibrotic and cirrhotic liver but not in HCC, relative to healthy liver. (Kruskal–Wallis test with Dunn's post-hoc, overall difference among groups ($H(3)$ = 92.31, p<0.0001). Significant pairwise differences: Normal and Fibrosis (p<0.0001) and Normal and HCC (p<0.0001); Normal vs Cirrhosis was not significant.

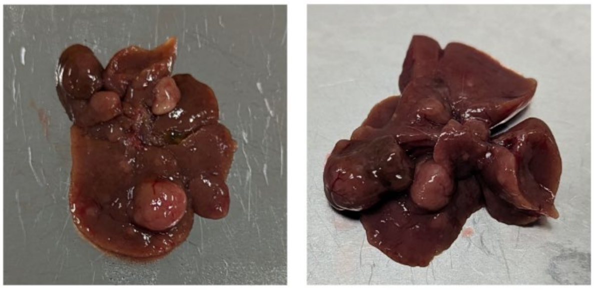

Figure S10: Photographs of Mdr2$^{-/-}$ mouse livers

Representative photographs of whole livers from 11 month old male Mdr2$^{-/-}$ mice, showing multiple large hepatocellular carcinomas visible on external surfaces of the liver.

a Image showing region-of-interest analysis for individual liver tumors of Mdr2$^{-/-}$ mice

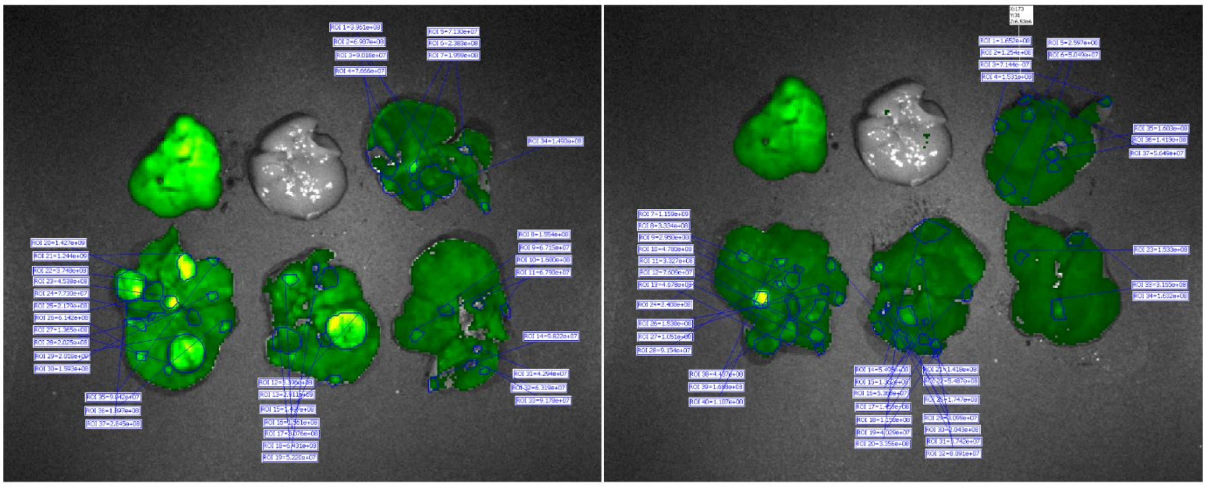

b eGFP fluorescence in livers and individual tumors of Mdr2$^{-/-}$ mice

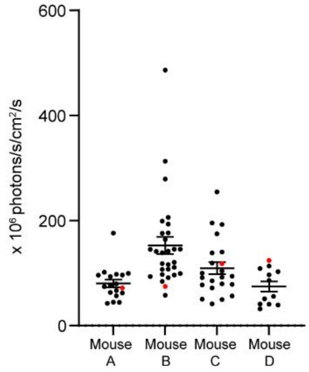

Figure S11: Region-of-interest analysis of individual tumors of Mdr2$^{-/-}$ mice

(a) To analyse the fluorescence of individual liver tumors from Mdr2$^{-/-}$ mice, regions of interest were drawn around identifiable tumors on both sides of the livers using the LivingImage software package. **(b)** Fluorescence of each region was calculated for each of four mice. The red point in each column represents the average fluorescence of the entire liver, including all tumors.

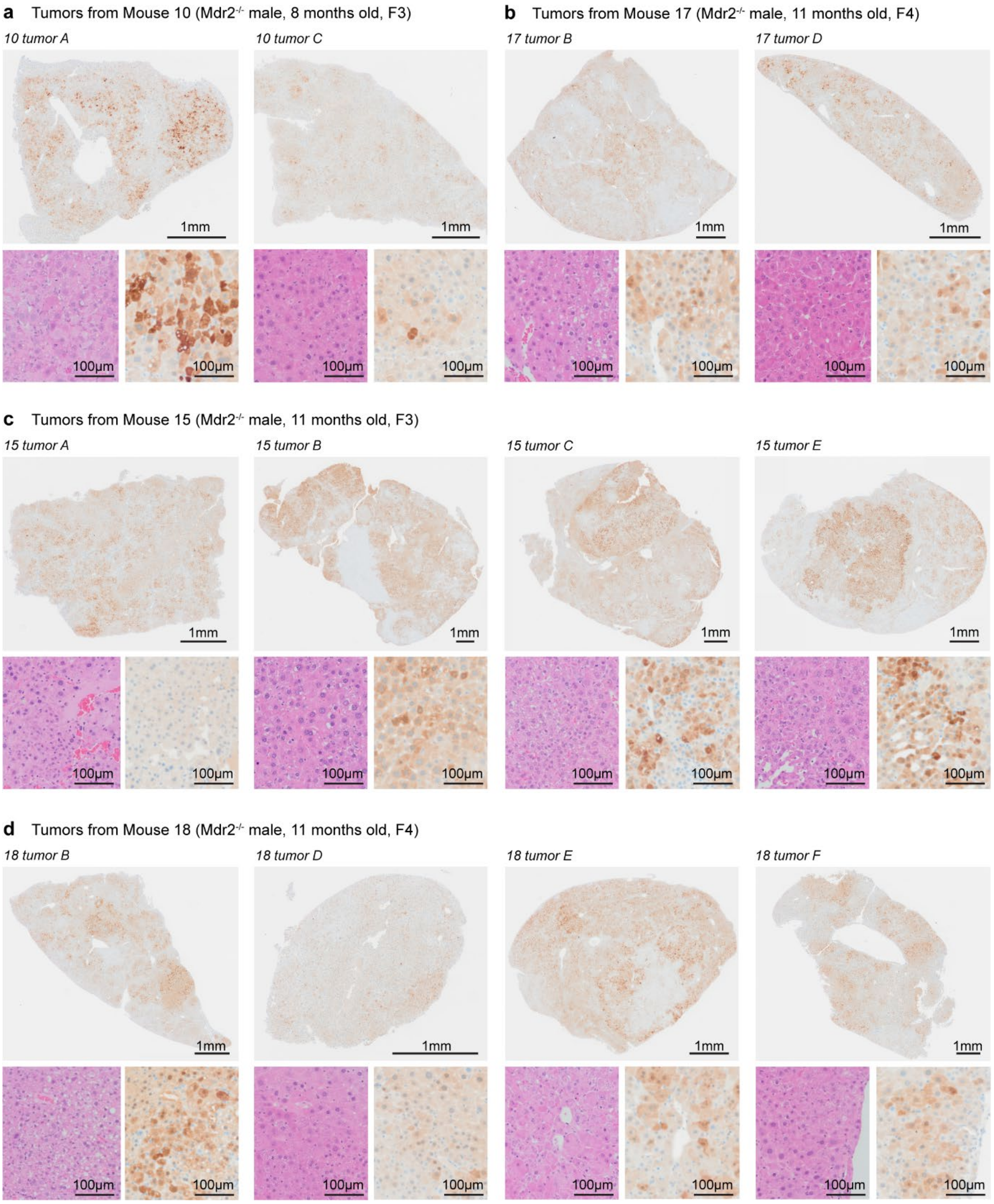

Figure S12: Additional histology images, Mdr2$^{-/-}$ HCCs

Mdr2$^{-/-}$ mice develop numerous HCCs with age. These images show the range of outcomes for mRNA-LNP delivery to various HCCs from four different male Mdr2$^{-/-}$ mice.

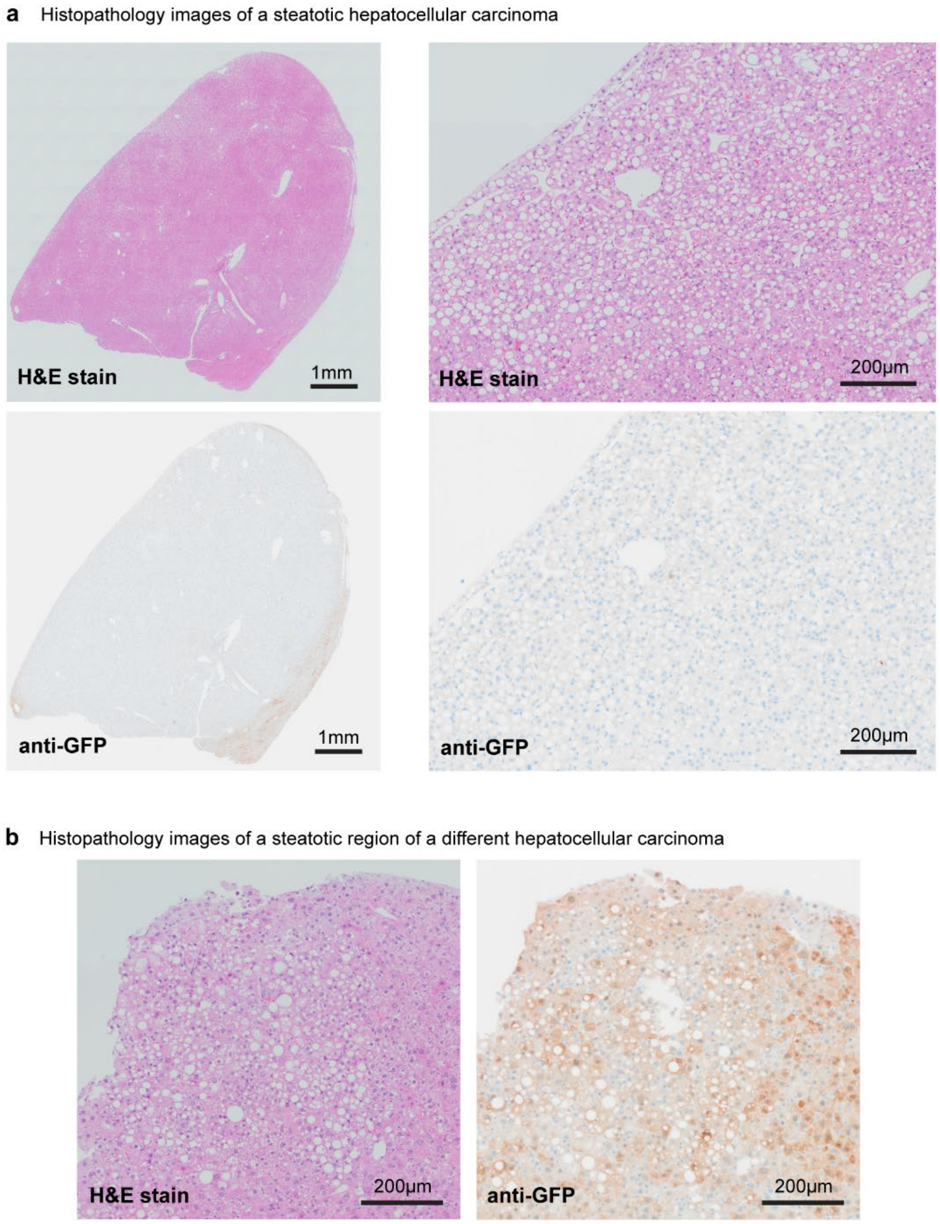

Figure S13: Delivery of mRNA-LNPs to steatotic HCC

(a) mRNA-LNP delivery to a steatotic HCC from an 11 month old male Mdr2$^{-/-}$ mouse was notably poor, with eGFP expression only observed in a narrow band around the edge of the tumor. **(b)** Presence of steatosis is compatible with good mRNA-LNP delivery and expression, as demonstrated by strong eGFP expression in a steatotic region of a different HCC.

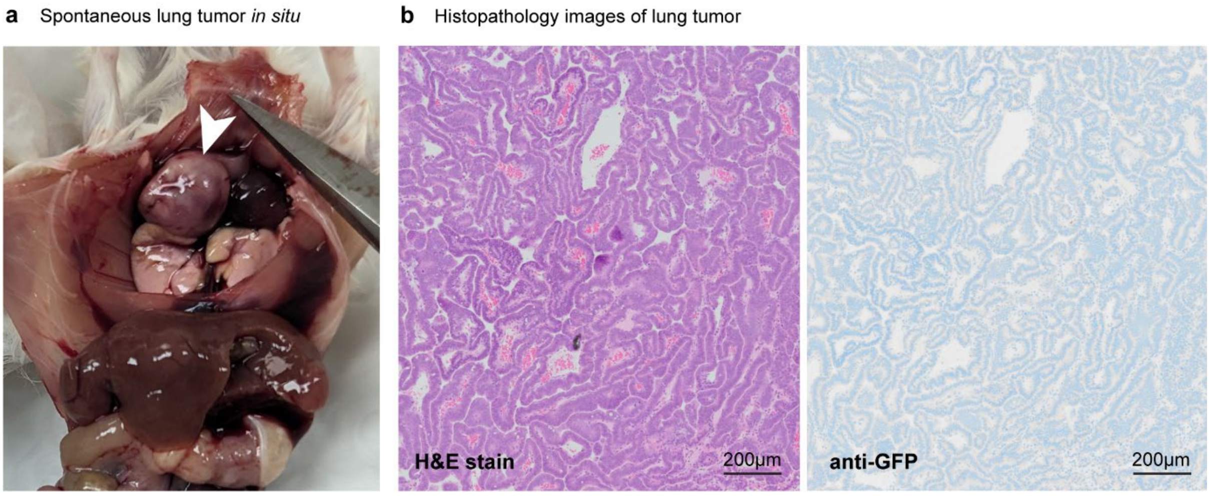

Figure S14: mRNA-LNPs were not delivered to one lung adenocarcinoma

(a) A large, spontaneous adenocarcinoma of the lung was recovered from an 11 month old male Mdr2$^{-/-}$ mouse.
(b) Delivery of mRNA-LNPs to this tumor was negligible, with eGFP staining not detectable above background.

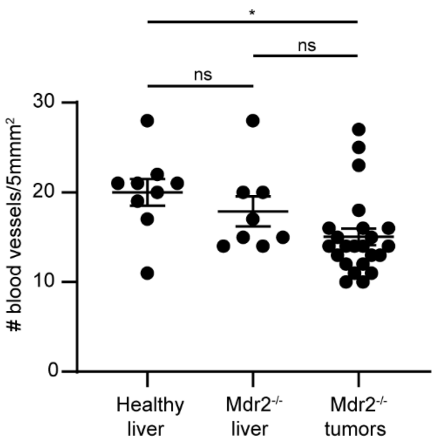

Figure S15: Comparison of blood vessel count in healthy and Mdr2$^{-/-}$ animals

The vascularity of liver tissue and tumors was assessed by counting the number of blood vessels in 5mm2 of tissue (i.e., 5 fields-of-view at 20X magnification). A significant difference was observed between healthy liver and the tumors of Mdr2$^{-/-}$ animals. (Kruskal–Wallis test with Dunn's post-hoc, *o*verall difference among groups ($H(2)=9.025$, p=0.0110). Dunn's post-hoc comparisons: Healthy liver vs Mdr2$^{-/-}$ liver, ns (p>0.9999; n=8-9); Healthy liver vs Mdr2$^{-/-}$ tumors, * (p=0.0131, n=9-23); Mdr2$^{-/-}$ liver vs Mdr2$^{-/-}$ tumors, ns (p=0.2769; n=8-23).

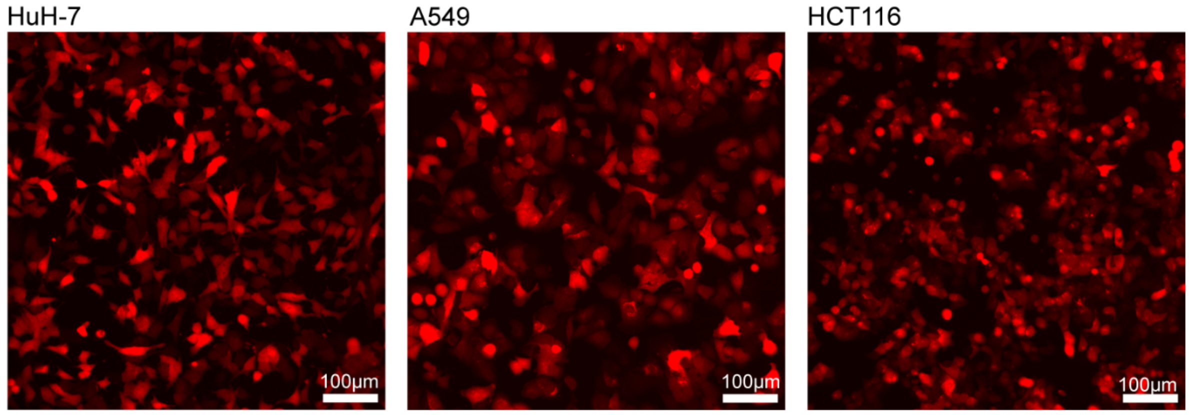

Figure S16: Demonstration of mCherry expression in cell lines

Fluorescence images showing strong expression of mCherry in knock-in cell lines used for animal experiments.

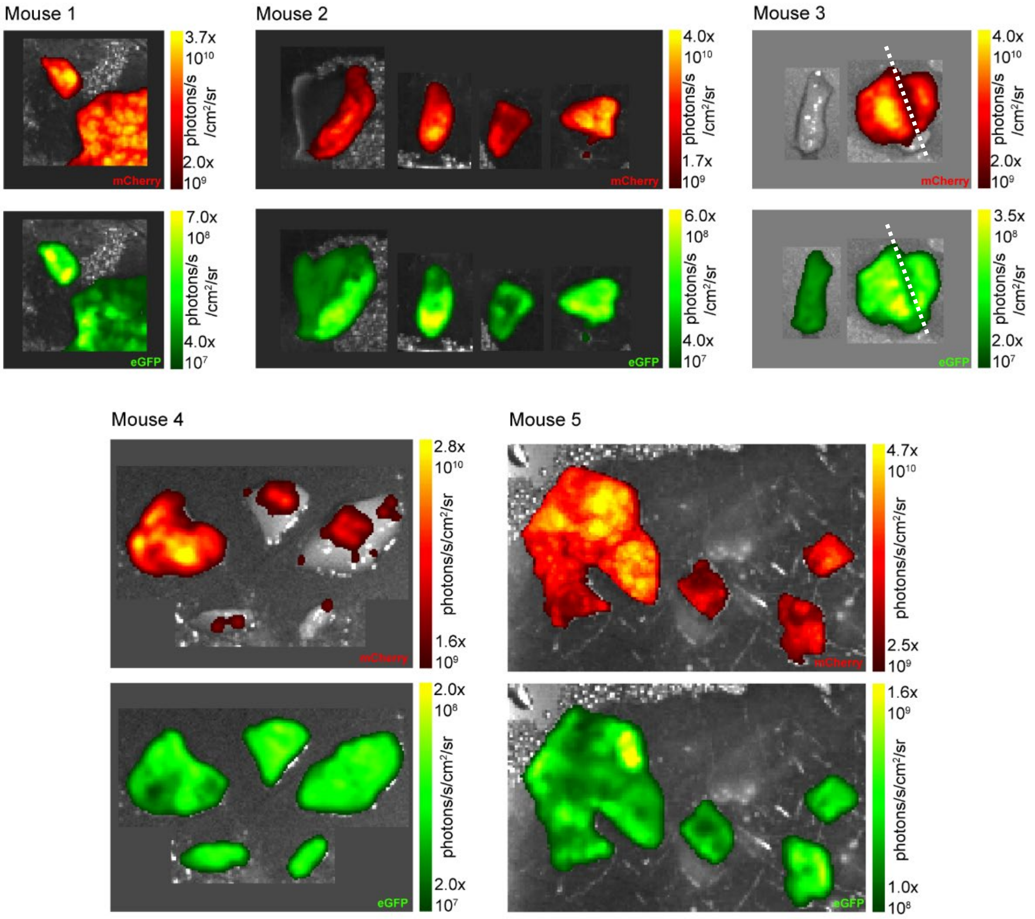

Figure S17: Fluorescence images for internal regions of liver tumors derived from xenografted HuH-7 cells

Ex vivo fluorescence imaging of liver tumors derived from HuH-7 human hepatocellular carcinoma cells xenografted into BALB/c nude mice. During tissue collection and imaging, tumors were cut into multiple pieces to provide samples for multiple analytical techniques, and images were taken to verify tissue identity and evenness of eGFP expression in internal regions of the tumors. Red (mCherry) identifies tumors, green identifies eGFP. Images show small sections of HuH-7 derived tumors with cut surfaces facing the camera. In most examples, even fluorescent signal is observed across the cut edge of the tumor, illustrating that eGFP is present throughout the tumor and not merely at the margins or in overlying healthy liver tissue.

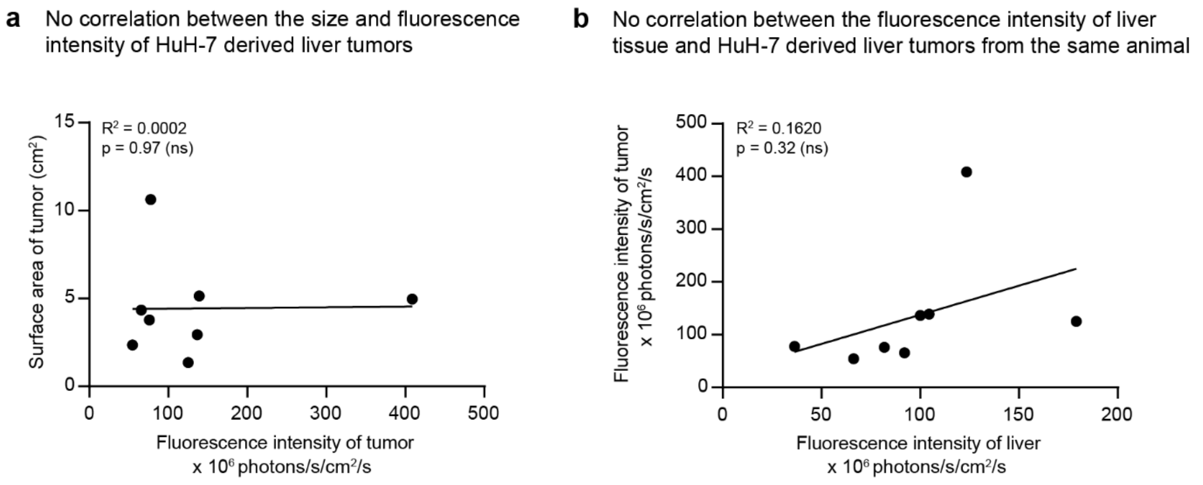

Figure S18: Additional analysis of fluorescence imaging data for HuH-7 derived xenografts

(a) There is no correlation between the size of HuH-7 derived liver tumors and their fluorescence intensity (Simple linear regression, slope not significantly different from zero (p=0.97)). **(b)** In mice bearing HuH-7 liver xenografts, there is no correlation between the fluorescence intensity of the liver tissue and the HuH-7 derived tumor (Simple linear regression, slope not significantly different from zero (p=0.1620)).

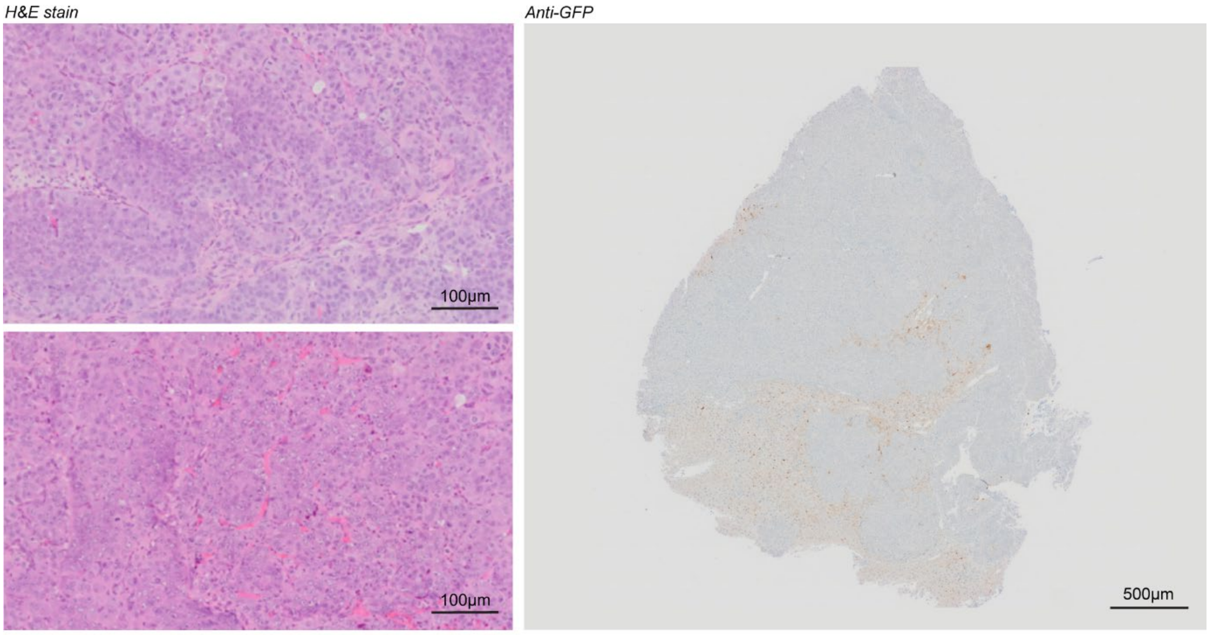

Figure S19: Additional histology images for HuH-7 derived xenograft liver tumor

H&E stain from representative regions of the tumor demonstrates tissue architecture. Anti-GFP stain shows low-level expression of eGFP throughout the tumor, indicating successful delivery of mRNA-LNPs. Note the presence of residual hepatocytes/liver tissue at the lower margin of the tumor. Smaller cells and higher cell density within the xenograft relative to the healthy liver makes the tumor tissue appear more blue; the colour of the cytoplasm (indicating eGFP staining) is comparable.

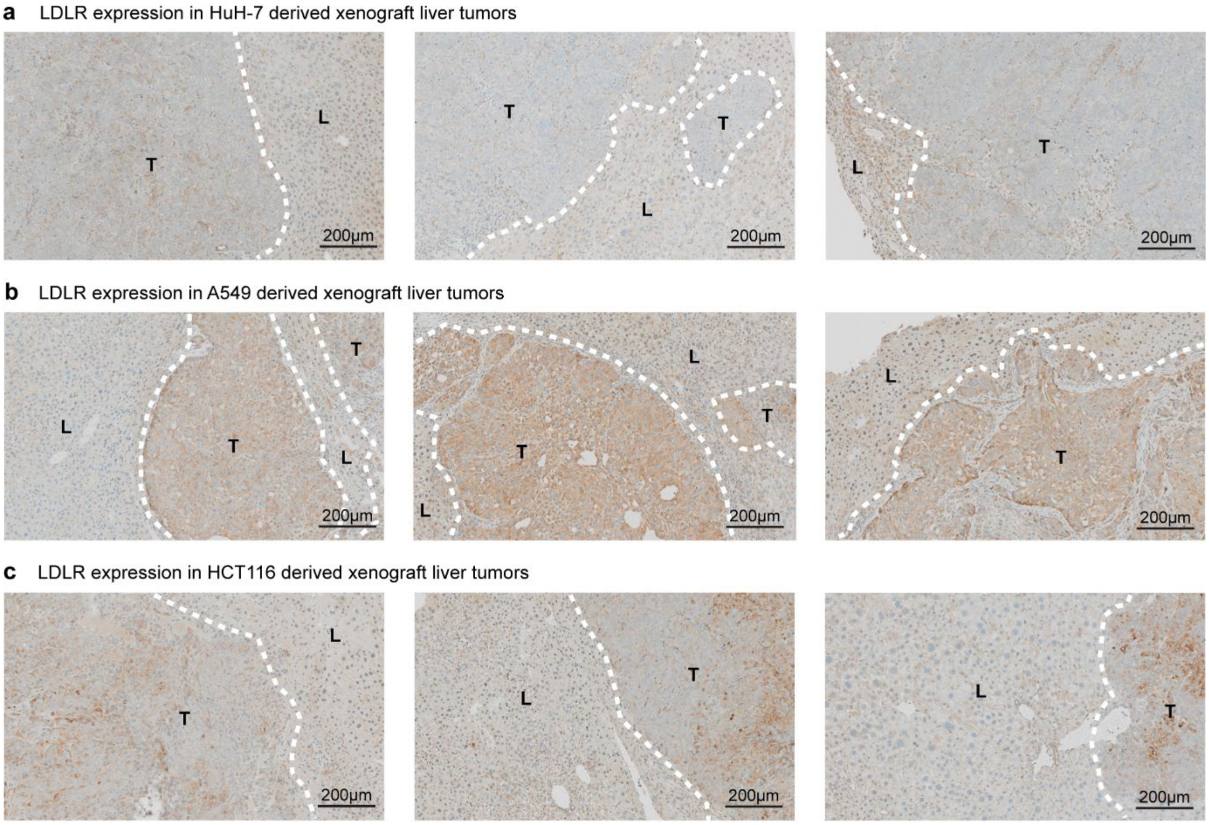

Figure S20: LDLR expression in xenograft-derived liver tumors

LDLR expression was examined by IHC in liver and tumor sections from 3 representative mice bearing tumors derived from xenografted HuH-7 **(a)**, A549 **(b)** and HCT116 **(c)** cells. Moderate membrane-enhanced expression of LDLR was observed in the liver tissue from all mice. LDLR expression in the tumors is broadly comparable to the liver tissue in HuH-7 tumors **(a)**, stronger relative to the liver tissue in A549 tumors **(b)**, and comparable to the liver tissue with some hyperintense cells in HCT116 tumors **(c)**. Note that the sections were stained using a primary antibody which recognises both human (in the xenografts) and mouse (in the liver parenchyma) LDLR sequences, but which may not have recognised both with equal affinity.

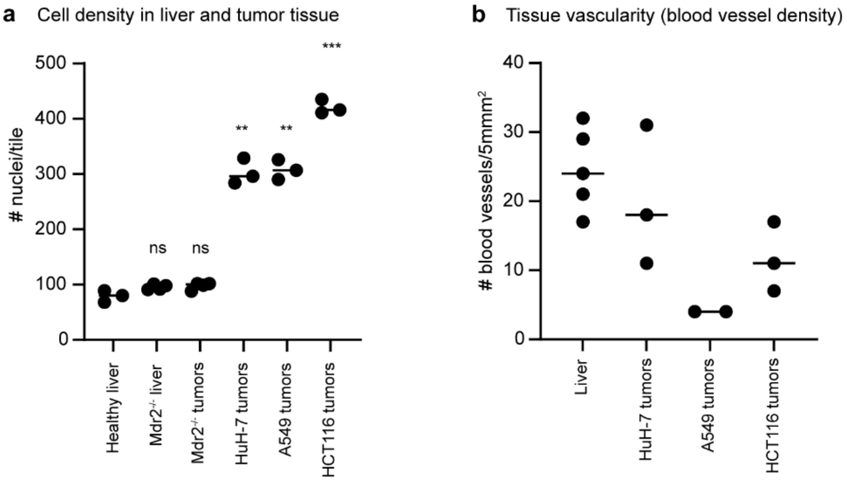

Figure S21: Cell density and tissue vascularity in xenograft-derived liver tumors

(a) As a proxy for cell density/size, we counted nuclei in one 500*500 pixel field of view for each of 3 representative tissue sections for liver tissue from healthy and Mdr2$^{-/-}$ mice, spontaneous HCCs of Mdr$^{-/-}$ mice, and xenografts derived from HuH-7, A549, and HCT116 cells. Brown–Forsythe and Welch ANOVA tests indicated a significant difference among groups ($F^*(5,6.57)=334.3$, $p<0.0001$; Welch's $W(5,5.65)=325.2$, $p<0.0001$). Dunnett's T3 multiple comparisons test showed no significant difference between healthy liver and Mdr2$^{-/-}$ liver ($p=0.2595$) or between healthy liver and Mdr2$^{-/-}$ tumors ($p=0.2251$). In contrast, xenograft tumors derived from all three tested cell lines exhibited significantly higher cell densities than healthy liver, including HuH-7 tumors ($p=0.0021$), A549 tumors ($p=0.0011$), and HCT116 tumors ($p<0.0001$). **(b)** As a proxy for tumor vascularity, we counted blood vessels in 5mm^2 of tissue (i.e., 5 fields of view at 20X magnification). Due to low n, statistical analysis was not performed. Qualitatively, vascularity of HuH-7 derived xenografts appears comparable to healthy liver, while A549 and HCT116 appear to have lower blood vessel density.

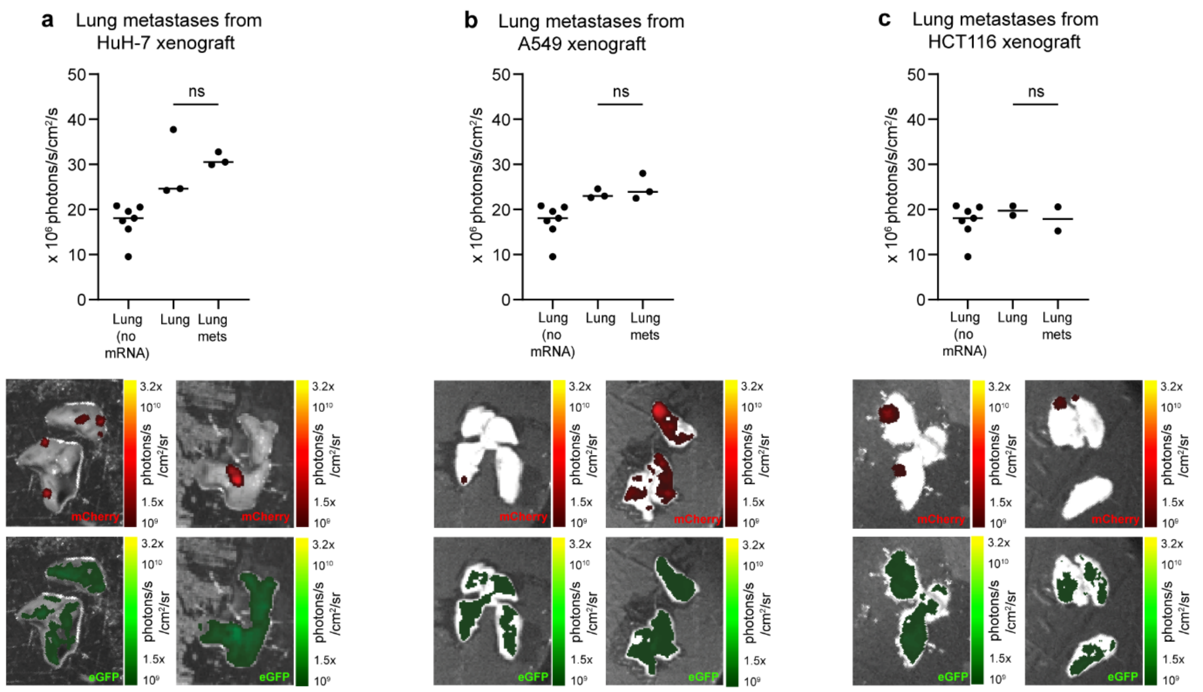

Figure S22: Fluorescence analysis of spontaneous lung metastases from mice bearing liver xenografts

In some animals bearing liver xenografts of human cancer cell lines, small metastases to the lungs were present (marked by red fluorescence). Region-of-interest analysis was used to compare the fluorescence of the metastases to the background fluorescence of the lung. There was no significant difference in fluorescence for any cell line, indicating that there is unlikely to be any substantial delivery of mRNA-LNPs to tumors of exogenous origin present in the lung. **(a)** No difference in fluorescence for HuH-7 derived lung metastases (Wilcoxon matched-pairs test, n=3, W=2, p=0.7500). **(b)** No difference in fluorescence for A549-derived lung metastases (Wilcoxon matched-pairs test, n=3, W=2, p=0.7500). **(c)** No difference in fluorescence for HCT116-derived lung metastases (Mann-Whitney test, n=2, U=1, p=0.6667).

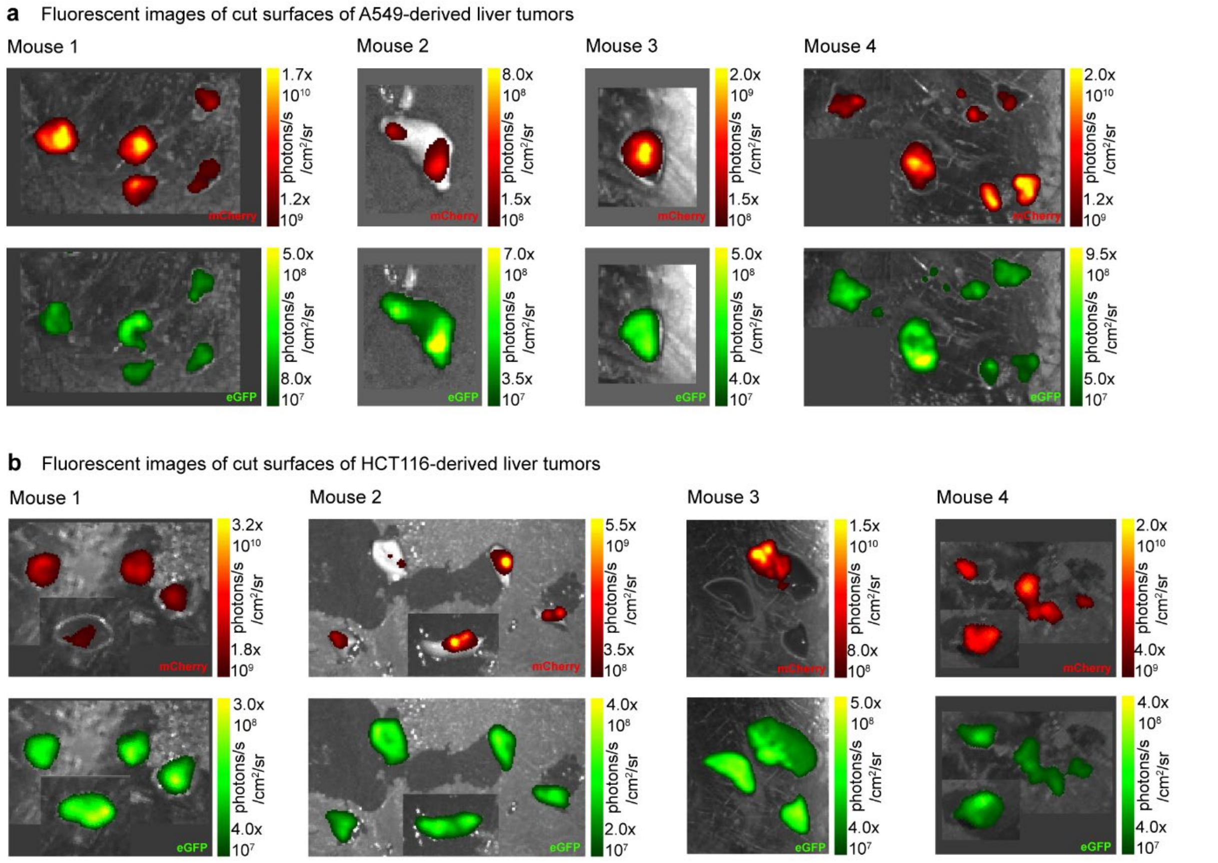

Figure S23: Fluorescence images for internal regions of liver tumors derived from A549 and HCT116 xenografts

Ex vivo fluorescence imaging of liver tumors derived from A549 **(a)** and HCT116 **(b)** cells xenografted into the livers of BALB/c nude mice. During tissue collection and imaging, tumors were cut into multiple pieces to provide samples for multiple analytical techniques, and images were taken to verify tissue identity and evenness of eGFP expression in internal regions of the tumors. Images show small sections of with cut surfaces facing the camera. In most examples, even fluorescent signal is observed across the cut edge of the tumor, illustrating that eGFP is present throughout the tumor and not merely at the margins or in overlying healthy liver tissue.

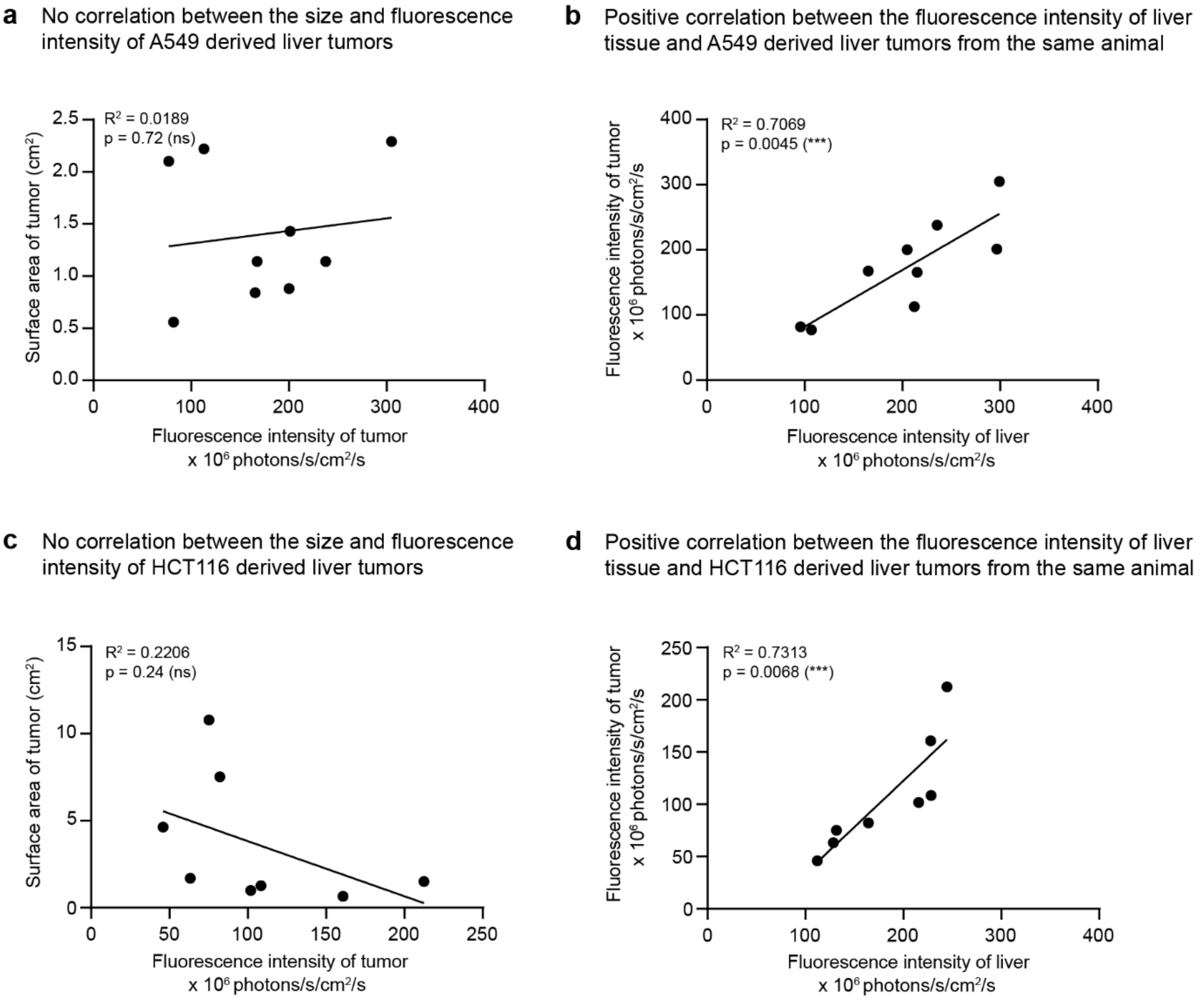

Figure S24: Additional analysis of fluorescence imaging data for A549 and HCT116 derived xenografts

(a) There is no correlation between the size of A549 derived liver tumors and their fluorescence intensity (Simple linear regression, slope not significantly different from zero (p=0.72)).
(b) The fluorescence intensity of liver tissue and A549 tumor tissue in the same animal is positively correlated (Simple linear regression, slope significantly different from zero (p=0.0045), 70.69% of the variance is explained by the linear model (R^2=0.7069)).
(c) There is no correlation between the size of HCT116 derived liver tumors and their fluorescence intensity (Simple linear regression, slope not significantly different from zero (p=0.24)).
(d) The fluorescence intensity of liver tissue and HCT116 tumor tissue in the same animal is positively correlated (Simple linear regression, slope significantly different from zero (p=0.0068), 73.13% of the variance is explained by the linear model (R^2=0.7313)).

a Additional histology images for representative liver tumor derived from A549 xenograft

H&E stain *Anti-GFP*

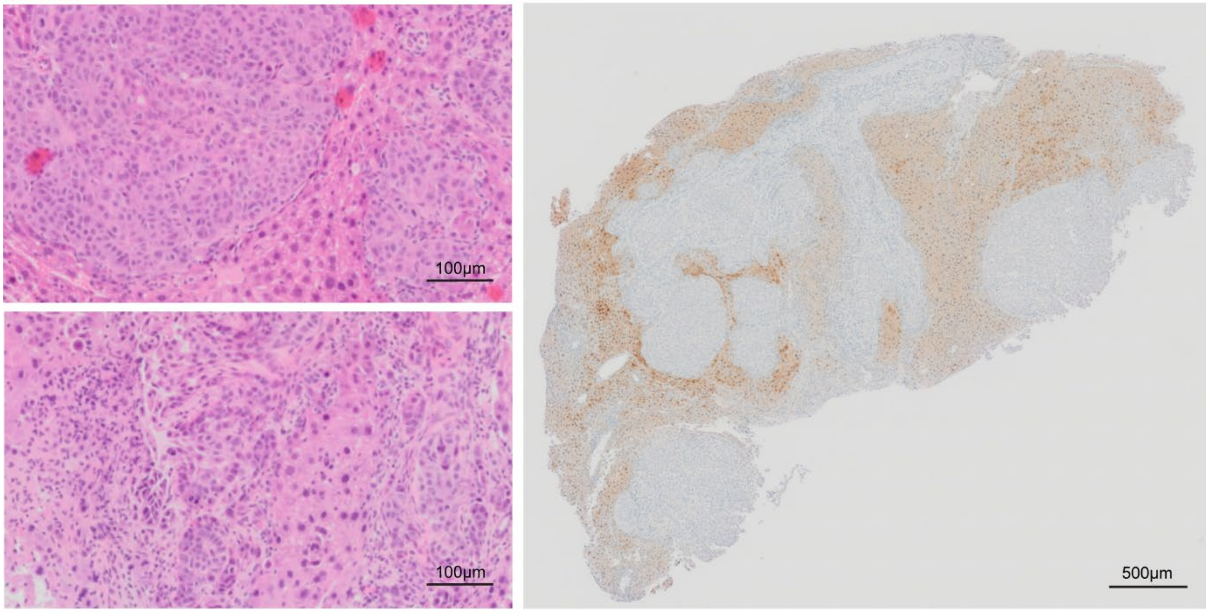

b Additional histology images for representative liver tumor derived from HCT116 xenograft

H&E stain *Anti-GFP*

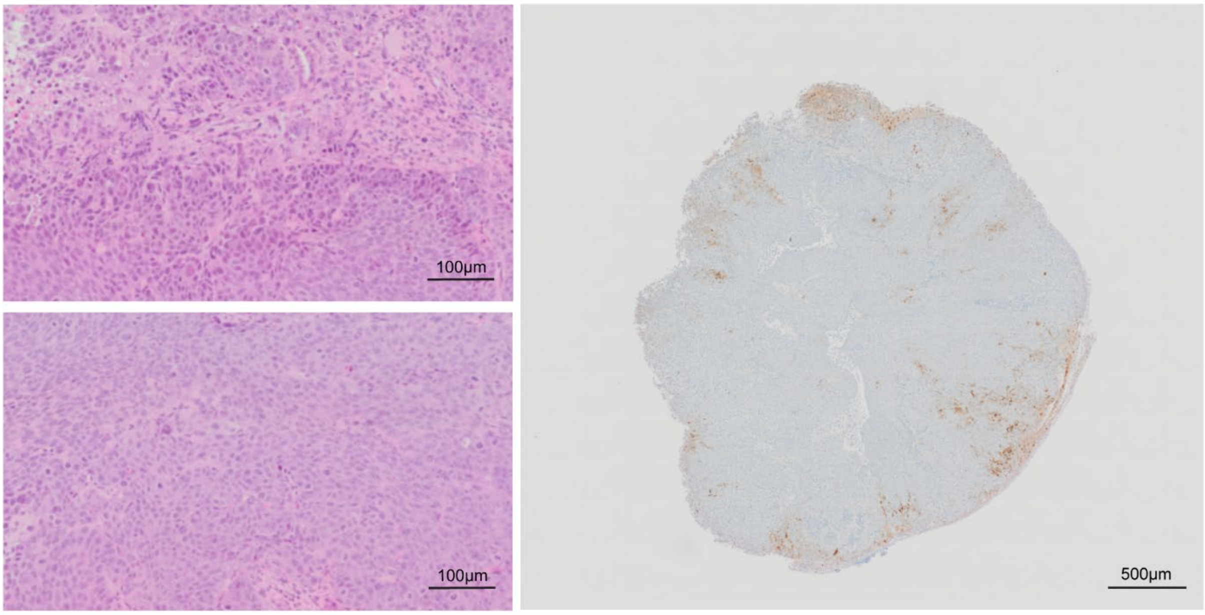

Figure S25: Additional histology images for liver xenografts modelling secondary liver cancer

H&E stain from representative regions of the tumor demonstrates tissue architecture. Anti-GFP stain shows location of eGFP delivery within the tumor. **(a)** liver tumor derived from A549 lung adenocarcinoma cells. **(b)** liver tumor derived from HCT116 colorectal carcinoma cells.

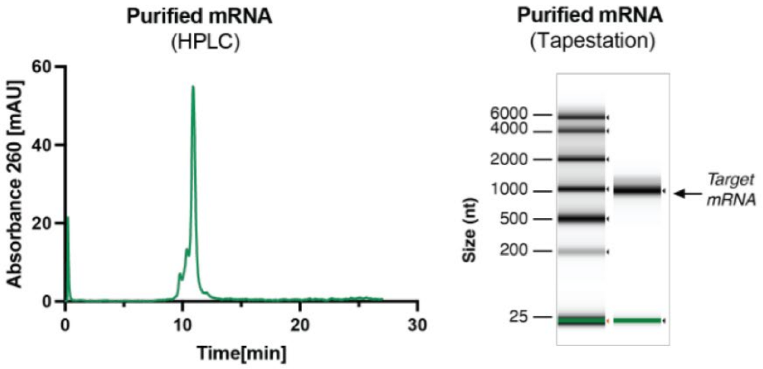

Analytical results for mRNA-LNPs (fresh formulation)

Analytical Property	Specification	Expected Specification	Result
**Size (nm)**	77.61	<100–200 nm	PASS
**PDI**	0.074	<0.3	PASS
**Zeta Potential (mV)**	2.964	+/-20 mV	PASS (*)
**Encapsulation Efficiency %**	98.0%	>80%	PASS

Analytical results for mRNA-LNPs (test thaw)

Analytical Property	Specification	Expected Specification	Result
**Size (nm)**	74.91	<100–200 nm	PASS
**PDI**	0.054	<0.3	PASS
**Zeta Potential (mV)**	3.256	+/-20 mV	PASS (*)

Figure S26: Excerpt from quality analysis of mRNA and mRNA-LNPs

Excerpt from a production report for a representative batch of eGFP mRNA and mRNA-LNPs, showing the expected size and purity of the synthetic mRNA, and the LNPs within specifications.

Table S1: Genes differentially regulated in liver tissue 24 hours after mRNA-LNP injection

Table S1 is supplied separately in .xlsx format.

Table S2: Antibody details

Application	Antibody	Host	Supplier	Cat#	Dilution
IHC-P	Anti-GFP	Rabbit	Novus	NB600-308	1:1000
IHC-P	Anti-LDLR	Rabbit	Thermo Fisher	10785-1-AP	1:500
Western blotting	Anti-GFP	Rabbit	Novus	NB600-308	1:2000
Western blotting	Anti-rabbit IgG, highly cross-adsorbed, Alexa Fluor™ Plus 800 conjugate	Donkey	Thermo Fisher	A32808	1:10000
